# Supplementary material for: Extracellular matrix sensing by FERONIA and Leucine‐Rich Repeat Extensins controls vacuolar expansion during cellular elongation in Arabidopsis thaliana
Source: EMBO J. 2019 Mar 8;38(7):e100353. doi: 10.15252/embj.2018100353 (PMC6443208; doi:10.15252/embj.2018100353)
Supplement: Supplementary file 1 — Appendix [file EMBJ-38-e100353-s001.pdf]

Table of contents

Appendix Figure S1. The cytosol shows relatively little increase during cellular elongation. 1

Appendix Figure S2. Fusicoccin rapidly acidifies the cell wall and affects vacuolar morphology. 2

Appendix Figure S3. FER-dependent signalling impacts on vacuolar size. 3

Appendix Figure S4. LRX1 - LRX7 show co-expression with FERONIA. 6

Appendix Figure S5. Redundancy of LRX3, LRX4 and LRX5 and cell size of *lrx3/4/5*. 7

Appendix Figure S6. FER and LRX reside in the same pathway. 9

Appendix Figure S7. Rapid RALF1 effect on apoplast pH. 10

Appendix Figure S8. FER co-immunoprecipitates with LRR4. 11

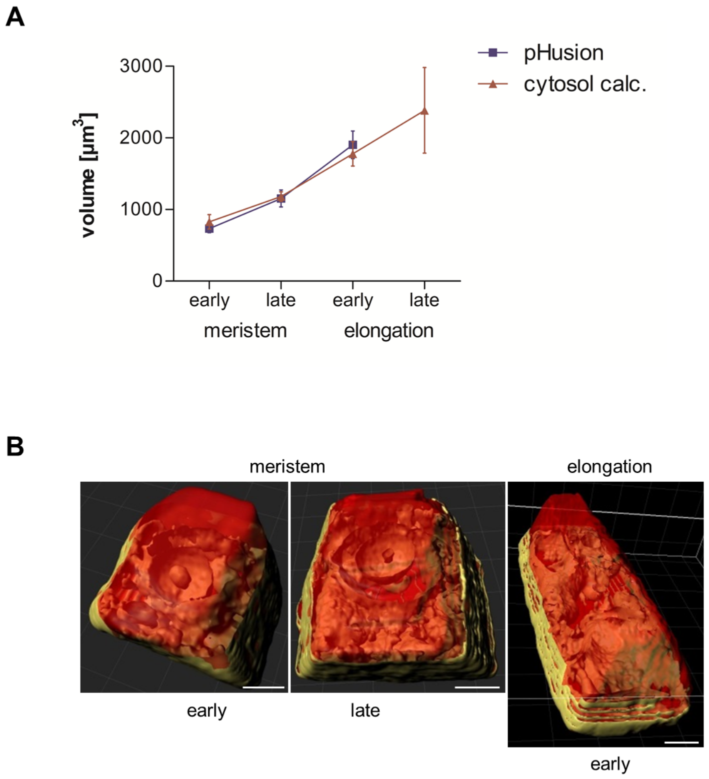

Appendix Figure S1. The cytosol shows relatively little increase during cellular elongation.

A Graph depicts measured (based on cytosolic fluorophore) and calculated (cytosol cal.) cytosol volumes of cells in the early and late meristem and in the early and late elongation zone. pHusion was used as a cytosolic fluorophore in early (n=6) and late meristematic (n=6) as well as early (n=5) elongation zone. Cytosol was also calculated, depicting the space between the vacuole and plasma membrane in early (n=7) and late (n=9) meristemic as well as early (n=11) and late (n=7) elongation zone. Data points represent mean with error bars depicting s.e.m.

B 3-D reconstructions of PI-stained cell walls (red) and pHusion-depicted cytosol (yellow) of cells in the early and late meristem and in the early elongation zone. Scale bars: 5 µm.

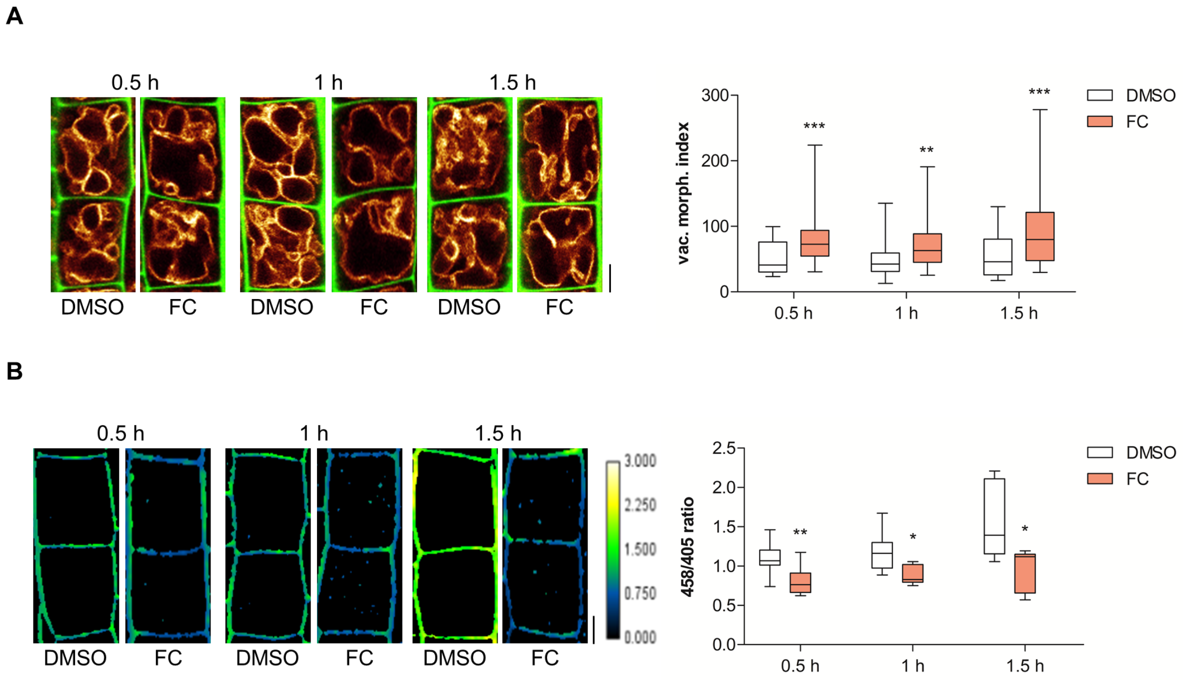

Appendix Figure S2. Fusicoccin rapidly acidifies the cell wall and affects vacuolar morphology.

A Representative images and quantification of vacuolar morphology of late meristematic cells. Cell walls were visualized with PI (green) and the vacuolar membrane with *pUBQ10::VAMP711*. Boxplot depicts vacuolar morphology index. Seedlings were treated with DMSO solvent control or 5 µM FC for 0.5 h (n=32), 1 h (n=32) or 1.5 h (n=28-32). Mann Whitney U test (**p < 0.01, ***p < 0.001).

B Representative color-coded images of cell walls of Col-0 after 0.5 h, 1 h and 1.5 h exposure to FC and quantification of the 458/405 ratio of HPTS in late meristematic cell walls (n=7-11). The apparent decrease in 458/405 ratio depicts cell wall acidification. Student´s *t*-test (*p < 0.05, **p < 0.01).

Data information: Scale bars: 5 µm. Boxplots: Box limits represent 25th and 75th percentile, horizontal line represents median. Whiskers display min. to max. values. Representative experiments are shown.

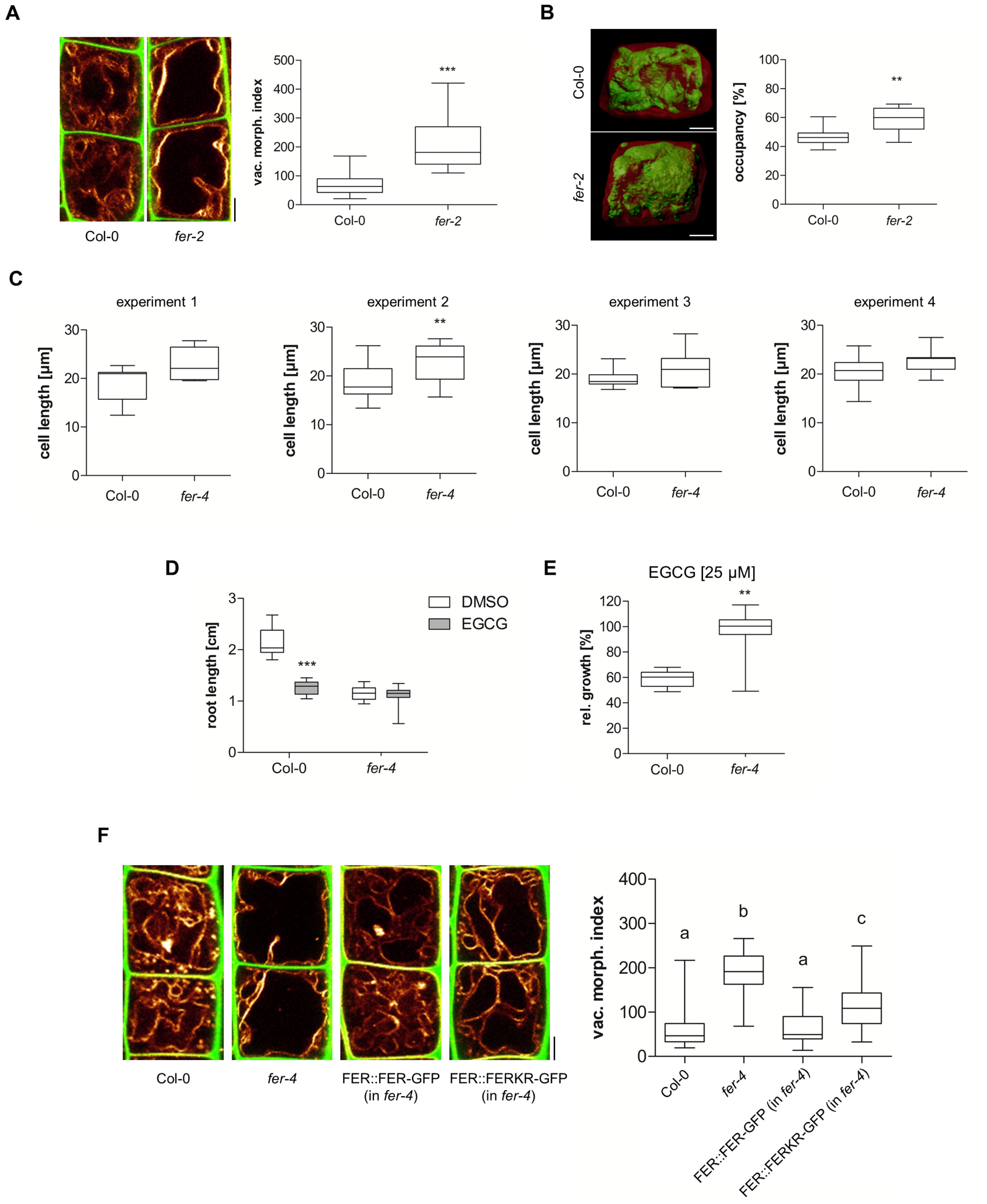

Appendix Figure S3. FER-dependent signalling impacts on vacuolar size.

A Representative images and quantification of vacuolar morphology of late meristematic atrichoblast cells of Col-0 (n=36) and *fer-2* (n=36). Mann Whitney U test (***p < 0.001). PI (green) and MDY-64 (yellow) staining depicts cell wall and vacuole, respectively.

B 3-D reconstructions of PI-stained cell wall (red) and BCECF-stained vacuole (green) of late meristematic atrichoblast cells of Col-0 (n=10) and *fer-2* (n=11). Student´s *t*-test (**p < 0.01). Boxplot depicts vacuolar occupancy of the cell.

C Average length of late meristematic atrichoblast cells in Col-0 and *fer-4* mutant background (n=8-14). Four independent experiments are shown. Student´s *t*-test (**p < 0.01).

D, E Absolute (D) and relative root length (E) of Col-0 (n=12) and *fer-4* (n=12) after 3 days of EGCG treatment. Student´s *t*-test (**p < 0.01, ***p < 0.001).

F Representative images and quantification of vacuolar morphology of late meristematic cells of Col-0 (n=32), *fer-4* (n=36), *pFER::FER-GFP* (n=36) in *fer-4* background and *pFER::FERKR-GFP* (n=36) in *fer-4* background. PI (green) and MDY-64 (yellow) staining depicts cell wall and tonoplast, respectively. Kruskal Wallis test followed by Dunn´s multiple comparison (b: p < 0.001, c: p < 0.01).

Data information: Scale bars: 5 µm. Boxplots: Box limits represent 25th and 75th percentile, horizontal line represents median. Whiskers display min. to max. values. Representative experiments are shown.

A

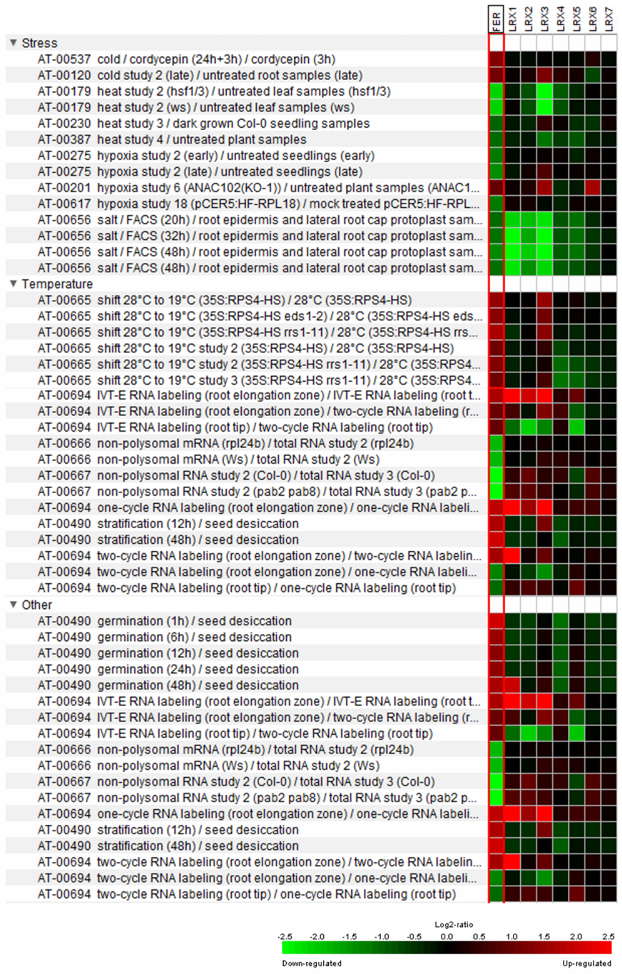
FER
LRX1
LRX2
LRX3
LRX4
LRX5
LRX6
LRX7
▼ Stress
AT-00537 cold / cordycepin (24h+3h) / cordycepin (3h)
AT-00120 cold study 2 (late) / untreated root samples (late)
AT-00179 heat study 2 (hsf1/3) / untreated leaf samples (hsf1/3)
AT-00179 heat study 2 (ws) / untreated leaf samples (ws)
AT-00230 heat study 3 / dark grown Col-0 seedling samples
AT-00387 heat study 4 / untreated plant samples
AT-00275 hypoxia study 2 (early) / untreated seedlings (early)
AT-00275 hypoxia study 2 (late) / untreated seedlings (late)
AT-00201 hypoxia study 6 (ANAC102(KO-1)) / untreated plant samples (ANAC1...
AT-00617 hypoxia study 18 (pCER5:HF-RPL18) / mock treated pCER5:HF-RPL...
AT-00656 salt / FACS (20h) / root epidermis and lateral root cap protoplast sam...
AT-00656 salt / FACS (32h) / root epidermis and lateral root cap protoplast sam...
AT-00656 salt / FACS (48h) / root epidermis and lateral root cap protoplast sam...
AT-00656 salt / FACS (48h) / root epidermis and lateral root cap protoplast sam...
▼ Temperature
AT-00665 shift 28°C to 19°C (35S:RPS4-HS) / 28°C (35S:RPS4-HS)
AT-00665 shift 28°C to 19°C (35S:RPS4-HS eds1-2) / 28°C (35S:RPS4-HS eds...
AT-00665 shift 28°C to 19°C (35S:RPS4-HS rrs1-11) / 28°C (35S:RPS4-HS rrs...
AT-00665 shift 28°C to 19°C study 2 (35S:RPS4-HS) / 28°C (35S:RPS4-HS)
AT-00665 shift 28°C to 19°C study 2 (35S:RPS4-HS rrs1-11) / 28°C (35S:RPS4...
AT-00665 shift 28°C to 19°C study 3 (35S:RPS4-HS rrs1-11) / 28°C (35S:RPS4...
AT-00694 IVT-E RNA labeling (root elongation zone) / IVT-E RNA labeling (root t...
AT-00694 IVT-E RNA labeling (root elongation zone) / two-cycle RNA labeling (r...
AT-00694 IVT-E RNA labeling (root tip) / two-cycle RNA labeling (root tip)
AT-00666 non-polysomal mRNA (rpl24b) / total RNA study 2 (rpl24b)
AT-00666 non-polysomal mRNA (Ws) / total RNA study 2 (Ws)
AT-00667 non-polysomal RNA study 2 (Col-0) / total RNA study 3 (Col-0)
AT-00667 non-polysomal RNA study 2 (pab2 pab8) / total RNA study 3 (pab2 p...
AT-00694 one-cycle RNA labeling (root elongation zone) / one-cycle RNA labeli...
AT-00490 stratification (12h) / seed desiccation
AT-00490 stratification (48h) / seed desiccation
AT-00694 two-cycle RNA labeling (root elongation zone) / two-cycle RNA labelin...
AT-00694 two-cycle RNA labeling (root elongation zone) / one-cycle RNA labeli...
AT-00694 two-cycle RNA labeling (root tip) / one-cycle RNA labeling (root tip)
▼ Other
AT-00490 germination (1h) / seed desiccation
AT-00490 germination (6h) / seed desiccation
AT-00490 germination (12h) / seed desiccation
AT-00490 germination (24h) / seed desiccation
AT-00490 germination (48h) / seed desiccation
AT-00694 IVT-E RNA labeling (root elongation zone) / IVT-E RNA labeling (root t...
AT-00694 IVT-E RNA labeling (root elongation zone) / two-cycle RNA labeling (r...
AT-00694 IVT-E RNA labeling (root tip) / two-cycle RNA labeling (root tip)
AT-00666 non-polysomal mRNA (rpl24b) / total RNA study 2 (rpl24b)
AT-00666 non-polysomal mRNA (Ws) / total RNA study 2 (Ws)
AT-00667 non-polysomal RNA study 2 (Col-0) / total RNA study 3 (Col-0)
AT-00667 non-polysomal RNA study 2 (pab2 pab8) / total RNA study 3 (pab2 p...
AT-00694 one-cycle RNA labeling (root elongation zone) / one-cycle RNA labeli...
AT-00490 stratification (12h) / seed desiccation
AT-00490 stratification (48h) / seed desiccation
AT-00694 two-cycle RNA labeling (root elongation zone) / two-cycle RNA labelin...
AT-00694 two-cycle RNA labeling (root elongation zone) / one-cycle RNA labeli...
AT-00694 two-cycle RNA labeling (root tip) / one-cycle RNA labeling (root tip)
Log2-ratio
-2.5 -2.0 -1.5 -1.0 -0.5 0.0 0.5 1.0 1.5 2.0 2.5
Down-regulated
Up-regulated

B

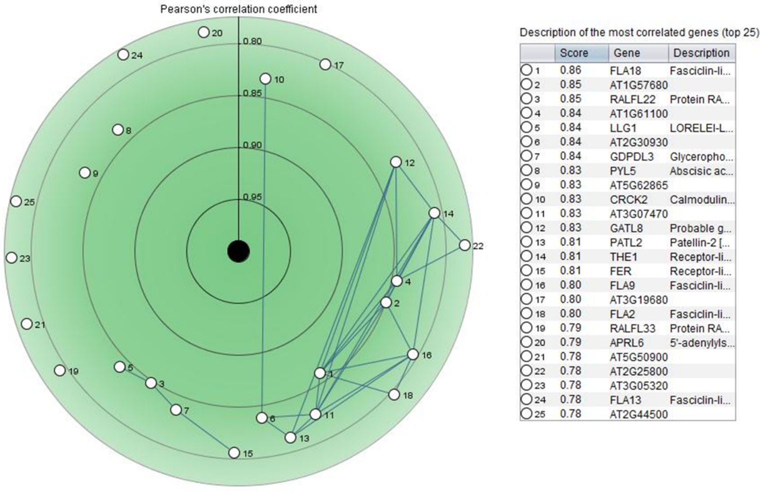
Pearson's correlation coefficient
0.80
0.85
0.90
0.95
Description of the most correlated genes (top 25)
Score Gene Description
1 0.86 FLA18 Fasciclin-li...
2 0.85 AT1G57680
3 0.85 RALFL22 Protein RA...
4 0.84 AT1G61100
5 0.84 LLG1 LORELEI-L...
6 0.84 AT2G30930
7 0.84 GDPDL3 Glycerophо...
8 0.83 PYL5 Abscisic ac...
9 0.83 AT5G62865
10 0.83 CRCK2 Calmodulin...
11 0.83 AT3G07470
12 0.83 GATL8 Probable g...
13 0.81 PATL2 Patellin-2 [...
14 0.81 THE1 Receptor-li...
15 0.81 FER Receptor-li...
16 0.80 FLA9 Fasciclin-li...
17 0.80 AT3G19680
18 0.80 FLA2 Fasciclin-li...
19 0.79 RALFL33 Protein RA...
20 0.79 APRL6 5'-adenylyls...
21 0.78 AT5G50900
22 0.78 AT2G25800
23 0.78 AT3G05320
24 0.78 FLA13 Fasciclin-li...
25 0.78 AT2G44500

Appendix Figure S4. LRX1 - LRX7 show co-expression with FERONIA.

A Correlation of *FER* expression with the vegetative clade of *LRXs (LRX1, LRX2, LRX3, LRX4, LRX5, LRX6, LRX7)* is shown upon perturbations (stress, temperature and other).

B List of genes that are co-expressed with LRX3 (correlation above 0.78) upon perturbations**.** Blue lines connect genes with a mutual correlation of at least 0.859.

Data information: Data was generated using Genevestigator. Expression data from the Affymetrix Arabidopsis ATH1 Genome Array was used and data with fold change = 2 and p-value < 0.01 was selected.

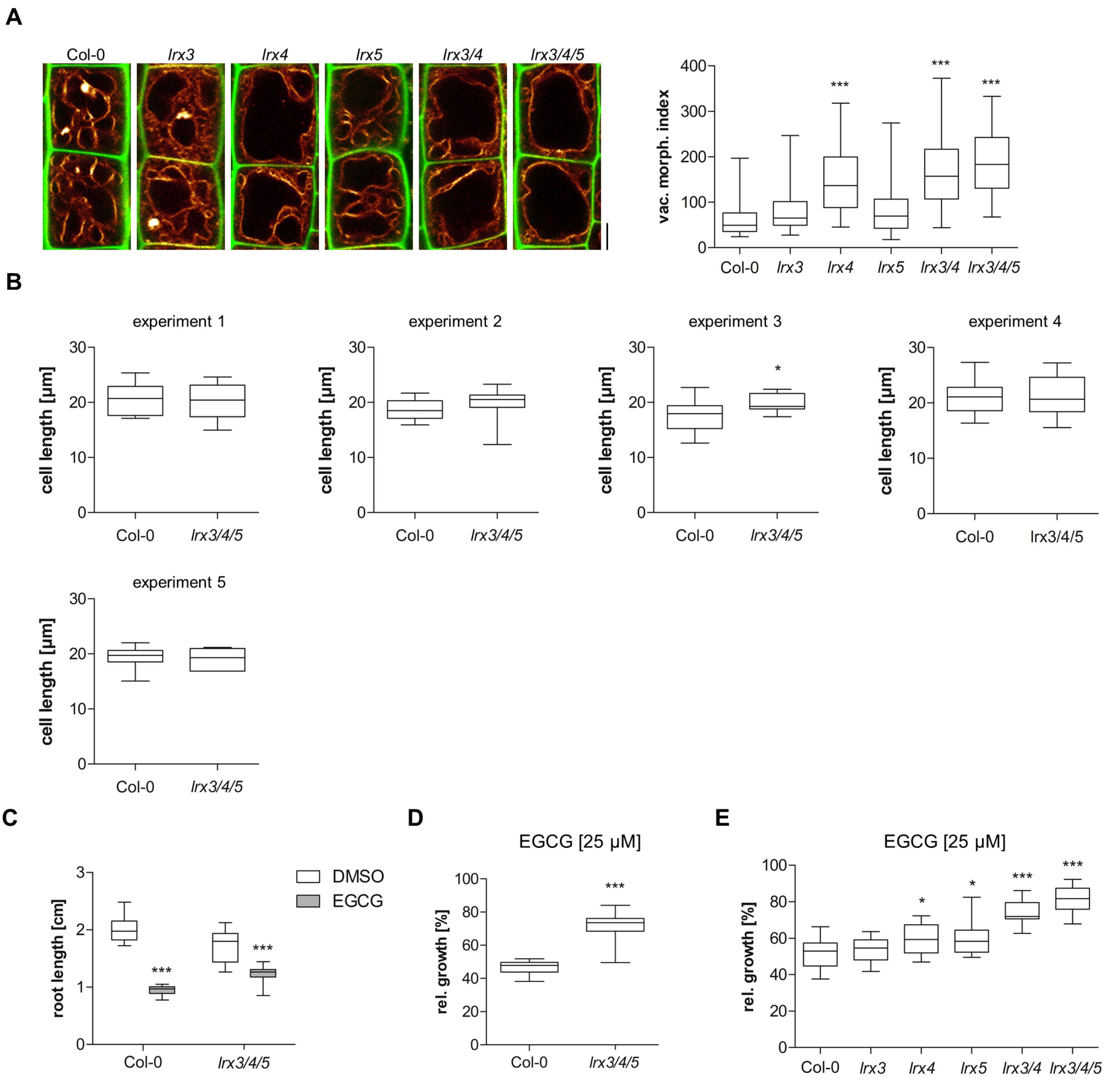

Appendix Figure S5. Redundancy of LRX3, LRX4 and LRX5 and cell size of *lrx3/4/5*.

A Representative images and quantification of vacuolar morphology of late meristematic cells of Col-0 (n=44), *lrx3* (n=36), *lrx4* (n=36), lrx5 (n=36), lrx3/4 (n=32) and *lrx3/4/5* (n=36). PI (green) and MDY-64 (yellow) staining depicts cell wall and tonoplast, respectively. Kruskal Wallis test followed by Dunn´s multiple comparison (***p < 0.001). Scale bar: 5 µm.

B Average length of late meristematic atrichoblast cells in Col-0 and *lrx3/4/5* mutant background (n=7-9). Five independent measurements are shown. Student´s *t*-test (*p < 0.05).

C, D Absolute (C) and relative root length (D) of Col-0 (n=12) and *lrx3/4/5* (n=12) after 3 days of EGCG treatment. Student´s *t*-test (***p < 0.001).

E Relative root length of Col-0 (n=35), *lrx3* (n=13), *lrx4* (n=11), lrx5 (n=12), lrx3/4 (n=12) and *lrx3/4/5* (n=12) after 3 days of EGCG treatment. One-way ANOVA followed by Bonferroni´s multiple comparison test (*p < 0.05, ***p < 0.001).

Data information: Boxplots: Box limits represent 25th and 75th percentile, horizontal line represents median. Whiskers display min. to max. values. Representative experiments are shown.

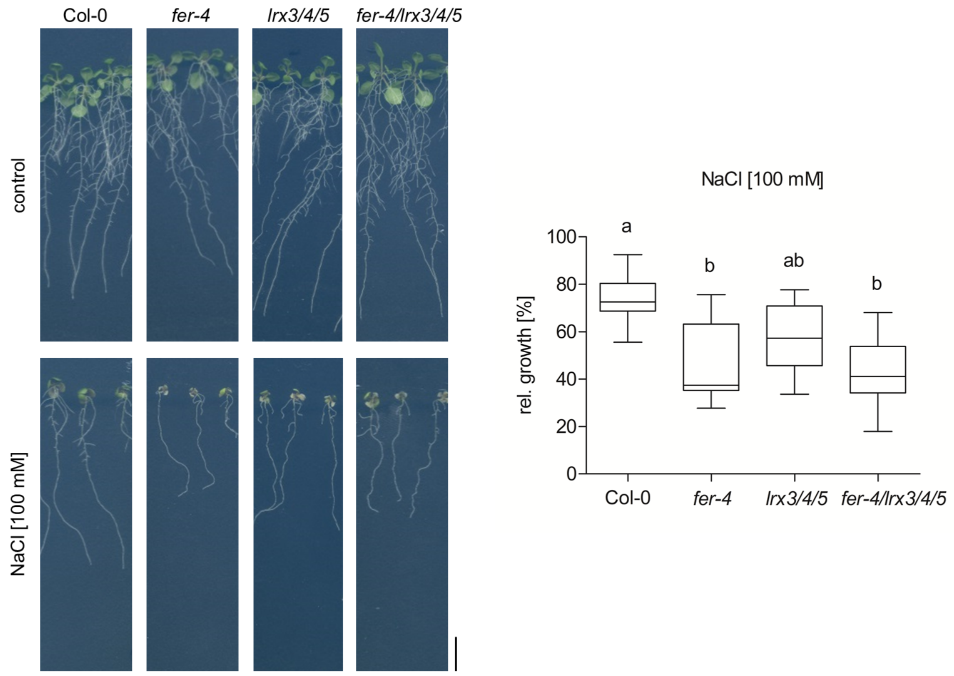

Appendix Figure S6. FER and LRX reside in the same pathway.

Scans of Col-0, *fer-4*, *lrx3/4/5* and *fer-4/lrx3/4/5* plantlets 10 days after transfer to NaCl supplemented or standard growth medium. Boxplot depicts relative root length of Col-0 (n=10), *fer-4* (n=6-9), *lrx3/4/5* (n=8-10) and *fer-4/lrx3/4/5* (n=7-9) on NaCl supplemented plates. One-way ANOVA followed by Bonferroni post test (b: $p < 0.001$; ab: not significantly different to a and b). Scale bar: 1 cm. Boxplots: Box limits represent 25th and 75th percentile, horizontal line represents median. Whiskers display min. to max. values. Representative experiments are shown.

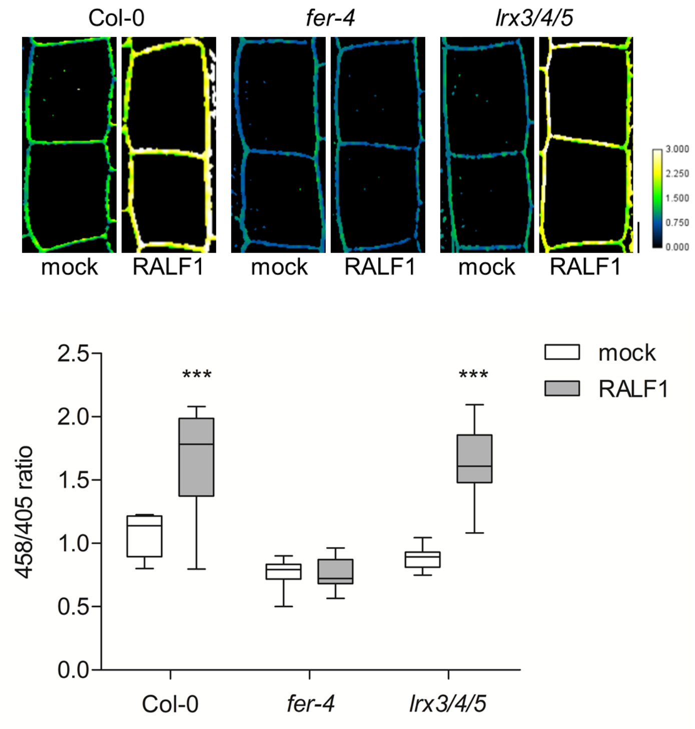

Appendix Figure S7. Rapid RALF1 effect on apoplast pH.

Representative color-coded images of cell walls of Col-0, *fer-4* and *lrx3/4/5* after 10 minutes exposure to 1 µM RALF1 and quantification of the 458/405 ratio of HPTS in late meristematic cell walls of the respective mutants (Col-0: n=10; *fer-4*: n=11; *lrx3/4/5*: n=11). Student´s *t*-test (***p < 0.001). Scale bar: 5 µm. Boxplots: Box limits represent 25th and 75th percentile, horizontal line represents median. Whiskers display min. to max. values. Representative experiments are shown.

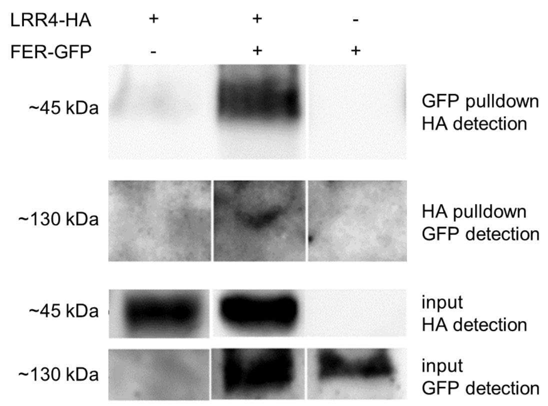

Appendix Figure S8. FER co-immunoprecipitates with LRR4.

LRR4-HA and FER-GFP were transiently expressed (as indicated by + and -). Immunoprecipitation and subsequent detection of the proteins by western blotting was done as labelled.
