# Supplementary material for: Extracellular matrix sensing by FERONIA and Leucine‐Rich Repeat Extensins controls vacuolar expansion during cellular elongation in Arabidopsis thaliana
Source: EMBO J. 2019 Mar 8;38(7):e100353. doi: 10.15252/embj.2018100353 (PMC6443208; doi:10.15252/embj.2018100353)
Supplement: Supplementary file 4 — Movie EV3 [file EMBJ-38-e100353-s004.zip › Movie_legend_EV3.docx]

**Movie EV3. 3D reconstruction of an atrichoblast cell in the early elongation zone.**

Vacuole is represented in green, the cell wall is displayed in red.
