# Supplementary material for: Extracellular matrix sensing by FERONIA and Leucine‐Rich Repeat Extensins controls vacuolar expansion during cellular elongation in Arabidopsis thaliana
Source: EMBO J. 2019 Mar 8;38(7):e100353. doi: 10.15252/embj.2018100353 (PMC6443208; doi:10.15252/embj.2018100353)
Supplement: Supplementary file 6 — Source Data for Appendix [file EMBJ-38-e100353-s013.zip › Figure_S7_Source_Data.pdf]

Appendix Figure S7

Col-0 control 458/405 ratio	average values/root	Col-0 RALF1 458/405 ratio	average values/root	*fer-4* control 458/405 ratio	average values/root	*fer-4* RALF1 458/405 ratio	average values/root	*lrx3/4/5* control 458/405 ratio	average values/root	*lrx3/4/5* RALF1 458/405 ratio	average values/root
0.798		0.712		0.726		0.921		0.893		0.953	
0.775		0.849		0.703		0.99		0.849		1.031	
0.802		0.827		0.713		0.986		0.919		1.165	
0.825		0.802		0.725		0.952		0.796		1.18	
	0.8		0.7975		0.71675		0.96225		0.86425		1.08225
0.922		1.049		0.833		0.803		0.927		1.482	
0.936		0.992		0.822		0.829		0.883		1.623	
0.919		1.077		0.785		1.003		0.876		1.625	
0.924		1.15		0.727		1.222		0.893		1.71	
	0.92525		1.067		0.79175		0.96425		0.89475		1.61
1.071		1.92		0.884		0.702		0.742		1.015	
1.123		1.874		0.9		0.685		0.675		1.315	
1.125		2.137		0.87		0.661		0.772		1.342	
1.211		1.999		0.952		0.684		0.81		1.336	
	1.1325		1.9825		0.9015		0.683		0.74975		1.252
0.621		2.075		0.748		0.687		0.892		1.556	
0.801		2.013		0.695		0.67		0.916		1.516	
0.913		1.922		0.733		0.703		1.03		1.434	
0.883		1.978		0.705		0.675		1.021		1.417	
	0.8045		1.997		0.72025		0.68375		0.96475		1.48075
1.146		1.415		0.776		0.787		0.848		1.238	
1.167		1.468		0.693		0.732		0.859		1.356	
1.131		1.423		0.811		0.774		0.888		1.609	
1.137		1.597		0.812		0.701		1		1.74	
	1.14525		1.47575		0.773		0.7485		0.89875		1.48575
1.134		1.564		0.814		0.546		0.74		1.271	
1.168		1.589		0.754		0.554		0.821		1.502	
1.129		1.84		0.858		0.572		0.756		1.607	
1.154		1.85		0.894		0.592		0.824		1.629	
	1.14625		1.71075		0.83		0.566		0.78525		1.50225
1.262		1.866		0.87		0.645		0.842		1.679	
1.142		1.811		0.826		0.744		0.869		1.816	
1.203		1.745		0.822		0.75		0.892		1.842	
1.29		1.546		0.82		0.681		0.861		2.08	
	1.22425		1.742		0.8345		0.705		0.866		1.85425
1.147		1.861		0.821		0.677		0.808		1.808	
1.238		1.925		0.773		0.743		0.821		1.979	
1.265		1.739		0.802		0.744		0.837		1.5	
1.206		1.765		0.796		0.726		0.776		1.667	
	1.214		1.8225		0.798		0.7225		0.8105		1.7385
1.176		2.067		0.623		0.63		0.872		1.517	
1.244		2.069		0.619		0.628		0.929		1.779	
1.154		2.064		0.694		0.606		0.908		1.793	
1.327		2.119		0.595		0.668		1.013		1.663	
	1.22525		2.07975		0.63275		0.633		0.9305		1.688
0.915		1.559		0.51		1.107		1.212		1.783	
0.871		1.947		0.509		0.769		1.008		1.793	
0.997		1.986		0.52		0.868		1.031		2.108	
1.162		2.238		0.463		0.744		0.932		2.361	
	0.98625		1.9325		0.5005		0.872		1.04575		2.01125
				0.76		0.782		1		2.407	
				0.886		0.696		0.909		1.908	
				0.868		0.727		0.76		1.912	
				0.916		0.757		0.905		2.153	
					0.8575		0.7405		0.8935		2.095
