# Supplementary material for: Extracellular matrix sensing by FERONIA and Leucine‐Rich Repeat Extensins controls vacuolar expansion during cellular elongation in Arabidopsis thaliana
Source: EMBO J. 2019 Mar 8;38(7):e100353. doi: 10.15252/embj.2018100353 (PMC6443208; doi:10.15252/embj.2018100353)
Supplement: Supplementary file 6 — Source Data for Appendix [file EMBJ-38-e100353-s013.zip › Figure_S6_Source_Data.pdf]

Appendix Figure S6

Col-0 NaCl relative	*fer-4* NaCl relative	*lrx3/4/5* NaCl relative	*fer-4/lrx3/4* relative
69.03856	69.89555	64.98174	56.14461
70.98151	56.43531	42.37504	68.08009
74.15075	37.2473	46.74945	51.47277
70.96558	35.73113	54.76364	41.08907
79.31073	40.76819	59.8226	38.01854
67.65301	34.87197	76.90285	30.40823
82.35257	37.44946	68.80518	17.94449
92.51326	27.72911	53.71178	42.16211
55.61307	75.62332	77.70427	38.77792
79.75665		33.60952	
