# Supplementary material for: Extracellular matrix sensing by FERONIA and Leucine‐Rich Repeat Extensins controls vacuolar expansion during cellular elongation in Arabidopsis thaliana
Source: EMBO J. 2019 Mar 8;38(7):e100353. doi: 10.15252/embj.2018100353 (PMC6443208; doi:10.15252/embj.2018100353)
Supplement: Supplementary file 6 — Source Data for Appendix [file EMBJ-38-e100353-s013.zip › Figure_S1_Source_Data.pdf]

Appendix Figure S1

cytopHusion

early meristem		late meristem		early elongation		late elongation	
cytopHusion	cytosol calc.	cytopHusion	cytosol calc.	cytopHusion	cytosol calc.		cytosol calc
948	1102	1161	1123.7	2598	2275.67		964
638	1194.98	1133	1022.016		1889.19		631
682	873.7	1156	1061.33	1489	1749		4100
846	492.22	1347	1611.7	1684	1323.97		2200
574	540.74	641	1253.3	1745	970		1600
718	875.59	1489	904.96	2000	1297.01		2300
	720.86		1380.7		2184.975		4894.45
			1123.7		2320.612		
					2248		
					2392.8		
					891.9232		
