# Supplementary material for: Extracellular matrix sensing by FERONIA and Leucine‐Rich Repeat Extensins controls vacuolar expansion during cellular elongation in Arabidopsis thaliana
Source: EMBO J. 2019 Mar 8;38(7):e100353. doi: 10.15252/embj.2018100353 (PMC6443208; doi:10.15252/embj.2018100353)
Supplement: Supplementary file 6 — Source Data for Appendix [file EMBJ-38-e100353-s013.zip › Figure_S3_Source_Data.pdf]

Appendix Figure S3A

	Col-0	vac. morph. index		*fer-2*	vac. morph. index
length	14.658		length	15.407	
width	7.961	116.6923	width	8.201	126.3528
	7.756			18.995	
	3.977	30.84561		14.727	279.7394
	9.895			18.337	
	7.2	71.244		18.034	330.6895
	8.406			11.775	
	6.492	54.57175		13.249	156.007
	15.974			13.065	
	8.257	131.8973		8.613	112.5288
	12.734			11.625	
	7.212	91.83761		9.441	109.7516
	12.121			14.939	
	5.335	64.66554		9.993	149.2854
	9.149			12.401	
	5.269	48.20608		8.841	109.6372
	10.23			16.825	
	8.285	84.75555		6.745	113.4846
	10.878			14.688	
	6.487	70.56559		9.373	137.6706
	13.448			12.263	
	6.663	89.60402		11.535	141.4537
	12.275			16.345	
	5.811	71.33003		10.812	176.7221
	6.126			16.578	
	5.097	31.22422		12.254	203.1468
	6.007			22.188	
	6.064	36.42645		14.904	330.69
	9.209			21.177	
	4.811	44.3045		14.466	306.3465
	8.44			15.379	
	4.72	39.8368		13.714	210.9076
	9.373			19.019	
	6.361	59.62165		15.23	289.6594
	12.244			15.865	
	5.851	71.63964		16.162	256.4101
	8.919			12.579	
	6.447	57.50079		9.529	119.8653
	5.068			13.489	
	4.113	20.84468		8.746	117.9748
	14.727			22.951	
	6.628	97.61056		18.317	420.3935
	11.485			18.626	
	6.693	76.86911		16.819	313.2707
	7.954			22.605	
	4.43	35.23622		15.184	343.2343
	6.732			17.226	
	4.811	32.38765		15.258	262.8343
	11.455			18.779	
	6.558	75.12189		13.489	253.3099
	11.31			19.295	
	3.73	42.1863		8.285	159.8591
	9.481			11.655	
	6.558	62.1764		12.487	145.536
	10.284			14.416	
	4.739	48.73588		13.954	201.1609
	15.874			17.644	
	10.615	168.5025		12.994	229.2661
	16.549			16.957	
	9.73	161.0218		14.184	240.5181
	14.506			18.525	
	9.956	144.4217		14.674	271.8359
	7.666			16.825	
	7.598	58.24627		9.924	166.9713
	14.208			12.845	
	6.32	89.79456		12.052	154.8079
	11.558			13.954	
	9.231	106.6919		10.812	150.8706
	9.505			13.215	
	4.835	45.95668		10.574	139.7354
	7.212			13.968	
	5.573	40.19248		13.321	186.0677

Appendix Figure S3B

Col-0 cell wall	vacuole	occupancy	*fer-2* cell wall	vacuole	occupancy
2636	1595	60.50835	5229	2858	54.65672
3407	1477	43.35192	4732	2254	47.63314
2525	1174	46.49505	3977	2066	51.94871
4457	2189	49.11375	8506	5889	69.23348
3290	1518	46.13982	5070	2173	42.85996
3900	1771	45.41026	7189	4511	62.74864
4224	2081	49.2661	4432	2952	66.6065
5667	3157	55.70849	4029	2437	60.48647
4174	1571	37.63776	6607	3957	59.89102
2263	966	42.6867	4289	2451	57.14619
2167	903	41.67051	4770	3168	66.41509

Appendix Figure S3C

Col-0		average cell length	*fer-4*		average cell length
length of 4 cells	83.109	20.77725	length of 4 cells	110.044	27.511
	82.892	20.723		74.963	18.74075
	90.58	22.645		93.224	23.306
	75.443	18.86075		87.842	21.9605
	88.419	22.10475		91.789	22.94725
	76.885	19.22125		92.744	23.186
	103.092	25.773		93.704	23.426
	74.482	18.6205		80.008	20.002
	57.428	14.357		93.224	23.306

Col-0		average cell length	*fer-4*		average cell length
length of 4 cells	72.339	18.08475	length of 4 cells	112.956	28.239
	92.507	23.12675		86.496	21.624
	75.684	18.921		69.677	17.41925
	71.601	17.90025		88.658	22.1645
	73.041	18.26025		68.476	17.119
	74.723	18.68075		81.213	20.30325
	67.274	16.8185		94.184	23.546
	80.729	20.18225		69.196	17.299

Col-0		average cell length	*fer-4*		average cell length
length of 4 cells	93.944	23.486	length of 4 cells	74.244	18.561
	104.778	26.1945		84.336	21.084
	97.795	24.44875		62.765	15.69125
	71.872	17.968		93.881	23.47025
	68.236	17.059		77.919	19.47975
	55.28	13.82		99.474	24.8685
	53.581	13.39525		102.596	25.649
	55.982	13.9955		100.431	25.10775
	69.452	17.363		110.539	27.63475
	76.885	19.22125		76.894	19.2235
	69.2	17.3		95.633	23.90825
	83.407	20.85175		106.875	26.71875
	69.928	17.482		106.444	26.611
	74.963	18.74075			

Col-0		average cell length	*fer-4*		average cell length
length of 4 cells	84.093	21.02325	length of 4 cells	94.425	23.60625
	85.054	21.2635		78.807	19.70175
	83.853	20.96325		93.703	23.42575
	60.547	15.13675		78.087	19.52175
	90.581	22.64525		82.892	20.723
	69.677	17.41925		111.004	27.751
	49.736	12.434		79.528	19.882
	84.575	21.14375		109.563	27.39075

Appendix Figure S3D and E

Col-0 DMSO		Col-0 EGCG 25µM		*fer-4* DMSO		*fer-4* EGCG 25µM	
absolute	relative	absolute	relative	absolute	relative	absolute	relative
2.192	102.4578	1.359	63.52199	0.947	82.69539	1.272	111.0755
1.984	92.73556	1.228	57.39882	1.16	101.2953	1.138	99.37418
1.943	90.81915	1.253	58.56737	1.173	102.4305	1.186	103.5657
2.089	97.64344	1.373	64.17637	1.048	91.51506	1.181	103.1291
2.443	114.19	1.327	62.02625	1.129	98.58827	0.563	49.16315
1.97	92.08117	1.375	64.26986	1.378	120.3318	1.16	101.2953
1.802	84.22857	1.044	48.79835	1.272	111.0755	1.341	117.1009
1.962	91.70724	1.056	49.35925	0.96	83.83059	1.122	97.977
2.675	125.0341	1.344	62.82086	1.148	100.2474	1.132	98.85024
2.645	123.6318	1.454	67.96245	1.272	111.0755	0.93	81.21089
1.883	88.01465	1.167	54.54758	1.031	90.03056		
2.085	97.45647	1.122	52.4442	1.224	106.884		

Appendix Figure S3F

Col-0			*fer-4*			pFER::FER-GFP (*fer-4*)			pFER::FERKR-GFP (*fer-4*		
		vac. morph.			vac. morph.			vac. morph.			vac. morph.
length	11.068	index	length	14.047	index	length	11.076	index	length	12.613	index
width	5.275	58.3837	width	12.073	169.5894	width	9.383	103.9261	width	5.934	74.84554
	12.542			13.697			7.421			12.686	
	5.159	64.70418		11.775	161.2822		4.733	35.12359		6.693	84.9074
	10.194			15.184			6.398			9.423	
	5.097	51.95882		12.606	191.4095		3.753	24.01169		10.637	100.2325
	6.007			16.834			7.998			7.903	
	3.799	22.82059		13.489	227.0738		6.126	48.99575		4.953	39.14356
	8.942			14.658			6.348			16.062	
	4.805	42.96631		15.167	222.3179		6.469	41.06521		10.376	166.6593
	8.409			23.307			10.812			11.734	
	4.325	36.36893		10.583	246.658		9.468	102.368		6.126	71.88248
	8.183			8.663			8.295			10.037	
	3.612	29.557		14.141	122.5035		5.588	46.35246		7.272	72.98906
	6.663			16.87			10.037			10.897	
	4.411	29.39049		9.602	161.9857		4.571	45.87913		11.97	130.4371
	19.028			18.741			12.461			15.865	
	11.397	216.8621		13.937	261.1933		7.737	96.41076		9.13	144.8475
	12.542			15.497			11.812			11.832	
	8.522	106.8829		7.452	115.4836		6.083	71.8524		9.196	108.8071
	15.845			12.531			8.893			12.851	
	8.938	141.6226		12.743	159.6825		5.63	50.06759		10.32	132.6223
	11.535			18.186			13.291			6.732	
	6.487	74.82755		14.591	265.3519		9.181	122.0247		5.696	38.34547
	11.655			17.195			9.006			14.161	
	5.068	59.06754		12.403	213.2696		4.865	43.81419		7.87	111.4471
	10.222			17.729			14.336			14.705	
	8.815	90.10693		10.539	186.8459		7.005	100.4237		9.373	137.83
	4.59			16.105			14.656			12.275	
	4.489	20.60451		12.754	205.4032		10.607	155.4562		6.007	73.73593
	5.399			16.34			5.097			11.485	
	3.547	19.15025		13.455	219.8547		3.398	17.31961		4.278	49.13283
	12.791			15.923			10.103			18.525	
	10.094	129.1124		14.208	226.234		6.727	67.96288		13.457	249.2909
	14.287			13.829			8.183			15.801	
	8.501	121.4538		15.497	214.308		7.464	61.07791		10.737	169.6553
	5.573			14.208			7.402			10.194	
	4.72	26.30456		13.455	191.1686		4.43	32.79086		10.583	107.8831
	9.133			11.861			3.761			11.333	
	4.865	44.43205		10.878	129.024		3.66	13.76526		8.437	95.61652
	9.373			15.384			11.094			11.117	
	5.051	47.34302		17.301	266.1586		7.756	86.04506		6.766	75.21762
	8.495			15.326			9.854			12.157	
	3.44	29.2228		11.254	172.4788		5.286	52.08824		8.596	104.5016
	6.88			16.964			9.863			8.603	
	4.859	33.42992		14.287	242.3647		4.571	45.08377		3.799	32.6828
	7.483			13.904			9.057			12.275	
	4.769	35.68643		13.532	188.1489		5.399	48.89874		8.201	100.6673
	9.044			17.059			7.903			18.352	
	6.872	62.15037		13.954	238.0413		5.399	42.6683		9.42	172.8758
	9.149			16.606			8.34			11.356	
	4.947	45.2601		12.291	204.1043		5.604	46.73736		9.718	110.3576
	8.34			13.448			11.055			10.836	
	3.911	32.61774		7.066	95.02357		5.286	58.43673		14.914	161.6081
	8.327			15.887			13.804			9.947	
	4.769	39.71146		13.217	209.9785		6.597	91.06499		5.198	51.70451
	16.098			16.825			11.622			12.035	
	9.37	150.8383		11.094	186.6566		11.605	134.8733		12.641	152.1344
	12.254			16.819			8.65			19.475	
	5.811	71.20799		13.457	226.3333		6.527	56.45855		11.533	224.6052
	10.334			14.951			9.271			14.424	
	6.011	62.11767		11.936	178.4551		6.398	59.31586		14.543	209.7682
	9.284			14.688			8.495			17.623	
	4.622	42.91065		11.315	166.1947		4.615	39.20443		7.856	138.4463
				15.137			15.093			13.217	
				11.655	176.4217		8.515	128.5169		7.483	98.90281
				19.865			7.417			15.444	
				11.535	229.1428		3.977	29.49741		7.224	111.5675
				15.839			6.007			14.102	
				7.272	115.1812		3.675	22.07573		8.706	122.772
				10.836			4.203			8.267	
				6.265	67.88754		4.278	17.98043		7.822	64.66447
