# Supplementary material for: Extracellular matrix sensing by FERONIA and Leucine‐Rich Repeat Extensins controls vacuolar expansion during cellular elongation in Arabidopsis thaliana
Source: EMBO J. 2019 Mar 8;38(7):e100353. doi: 10.15252/embj.2018100353 (PMC6443208; doi:10.15252/embj.2018100353)
Supplement: Supplementary file 6 — Source Data for Appendix [file EMBJ-38-e100353-s013.zip › Figure_S2_Source_Data.pdf]

Appendix Figure S2A

	0.5h DMSO	vac. morph. index	0.5h FC	vac. morph. index	1h DMSO	vac. morph. index	1h FC	vac. morph. index	1.5h DMSO	vac. morph. index	1.5h FC	vac. morph. index
length	9.499		16.162		9.158		7.066		12.431		10.823	
width	6.575	62.45593	11.861	191.6975	4.258	38.99476	7.815	55.22079	7.307	90.83332	6.745	73.00114
	6.895		16.354		9.133		8.977		15.379		9.623	
	4.008	27.63516	13.697	224.0007	4.947	45.18095	6.238	55.99853	7.598	116.8496	9.638	92.74647
	11.87		16.354		10.715		8.44		14.466		5.655	
	8.171	96.98977	12.368	202.2663	4.93	52.82495	6.702	56.56488	7.662	110.8385	5.247	29.67179
	6.347		12.736		9.37		7.2		10.331		8.073	
	4.587	29.11369	5.609	71.43622	7.932	74.32284	5.696	41.0112	6.05	62.50255	5.531	44.65176
	11.724		10.921		13.269		15.089		11.076		11.146	
	6.984	81.88042	6.758	73.80412	4.72	62.62968	12.641	190.74	4.9	54.2724	6.597	73.53016
	9.133		11.374		10.878		8.257		12.606		12.822	
	5.531	50.51462	6.585	74.89779	7.307	79.48555	7.636	63.05045	9.143	115.2567	6.083	77.99623
	7.958		10.274		9.209		10.942		11.795		12.809	
	4.865	38.71567	6.708	68.91799	4.59	42.26931	5.531	60.5202	6.984	82.37628	9.349	119.7513
	6.628		7.888		7.692		6.693		13.904		9.593	
	5.447	36.10272	5.166	40.74941	6.007	46.20584	6.732	45.05728	9.346	129.9468	7.632	73.21378
	13.602		10.357		12.815		8.13		7.958		18.567	
	6.469	87.99134	9.133	94.59048	4.665	59.78198	4.196	34.11348	4.953	39.41597	14.688	272.7121
	13.489		13.904		13.729		10.942		8.211		16.451	
	6.732	90.80795	5.934	82.50634	7.954	109.2005	8.423	92.16447	5.051	41.47376	15.089	248.2291
	13.328		9.149		8.368		10.401		5.731		12.878	
	5.934	79.08835	5.711	52.24994	5.447	45.5805	7.212	75.01201	3.941	22.58587	8.938	115.1036
	13.215		6.968		9.158		13.865		5.67		6.597	
	6.007	79.38251	4.384	30.54771	6.32	57.87856	7.212	99.99438	4.59	26.0253	5.811	38.33517
	8.812		7.323		14.291		10.365		11.555		12.795	
	6.026	53.10111	5.269	38.58489	4.953	70.78332	7.355	76.23458	5.771	66.68391	9.52	121.8084
	7.448		10.726		10.615		9.851		12.092		12.515	
	5.291	39.40737	12.18	130.6427	7.272	77.19228	7.005	69.00626	5.806	70.20615	11.292	141.3194
	7.525		10.812		6.32		10.583		7.212		15.619	
	4.028	30.3107	7.881	85.20937	4.787	30.25384	10.196	107.9043	4.59	33.10308	8.663	135.3074
	8.211		11.31		6.628		11.783		10.264		14.945	
	4.085	33.54194	6.011	67.98441	4.196	27.81109	8.257	97.29223	8.532	87.57245	7.005	104.6897
	9.65		10.037		6.804		9.999		7.521		10.194	
	4.085	39.42025	9.044	90.77463	5	34.02	7.005	70.043	6.732	50.63137	6.487	66.12848
	12.263		12.977		8.733		12.329		9.967		11.783	
	3.73	45.74099	7.929	102.8946	3.347	29.22935	6.007	74.0603	8.169	81.42042	9.243	108.9103
	5.335		10.572		9.271		10.103		9.149		9.877	
	5.247	27.99275	8.173	86.40496	3.753	34.79406	8.663	87.52229	6.558	59.99914	8.295	81.92972
	8.423		10.128		3.415		10.025		7.822		8.158	
	3.636	30.62603	5.934	60.09955	3.799	12.97359	5.63	56.44075	4.829	37.77244	3.874	31.60409
	7.881		12.312		5.275		14.561		5.318		17.554	
	5.437	42.849	9.993	123.0338	3.415	18.01413	9.933	144.6344	3.223	17.13991	9.383	164.7092
	8.413		9.019		4.59		11.533		6.265		13.628	
	5.308	44.6562	7.483	67.48918	3.738	17.15742	7.692	88.71184	4.028	25.23542	7.692	104.8266
	7.748		7.541		4.665		11.2		4.9		8.596	
	4.148	32.1387	6.8	51.2788	3.268	15.24522	5.291	59.2592	4.739	23.2211	6.645	57.12042
	7.87		10.014		8.864		9.209		7.692		11.295	
	3.911	30.77957	4.805	48.11727	4.769	42.27242	4.9	45.1241	3.398	26.13742	6.252	70.61634
	6.03		8.706		13.954		10.222		6.934		13.508	
	4.536	27.35208	6.126	53.33296	9.685	135.1445	6.14	62.76308	3.667	25.42698	6.252	84.45202
	6.575		14.01		6.238		12.734		5.655		11.169	
	3.927	25.82003	5.771	80.85171	5.479	34.178	10.103	128.6516	4.665	26.38058	6.984	78.0043
	6.752		9.181		8.423		7.903		6.252		8.285	
	3.439	23.22013	6.361	58.40034	5.479	46.14962	4.953	39.14356	3.675	22.9761	5.275	43.70338
	6.585		11.535		6.984		10.264		7.115		5.806	
	3.897	25.66175	6.732	77.65362	5.175	36.1422	3.874	39.76274	4.417	31.42696	5.811	33.73867
	10.929		8.938		7.212		6.154		9.478		20.831	
	5.934	64.85269	6.536	58.41877	5.286	38.12263	6.307	38.81328	8.169	77.42578	13.354	278.1772
	12.368		8.33		6.201		12.736		9.358		23.663	
	7.87	97.33616	5.479	45.64007	4.769	29.57257	6.766	86.17178	6.492	60.75214	7.703	182.2761
	10.103		15.645		7.034		7.228		5.816		6.026	
	9.854	99.55496	9.209	144.0748	6.05	42.5557	4.622	33.40782	5.108	29.70813	5.547	33.42622
	10.14		8.522		8.437		6.997		6.465		5.934	
	6.628	67.20792	7.421	63.24176	5.399	45.55136	3.636	25.44109	3.941	25.47857	5.846	34.69016

Appendix Figure S2B

0.5h DMSO

458/405 ratio	average values/root
0.984	
1.016	
1.021	
1.08	
	1.02525
0.873	
0.825	
0.602	
0.654	
	0.7385
1.094	
0.993	
1.045	
1.136	
	1.067
0.873	
1.003	
1.008	
1.184	
	1.017
0.993	
1.347	
1.224	
1.271	
	1.20875
1.155	
1.176	
1.308	
1.154	
	1.19825
1.105	
1.093	
1.133	
1.091	
	1.1055
1.479	
1.352	
1.511	
1.509	
	1.46275
0.946	
1.112	
0.932	
1.028	
	1.0045

0.5h FC

458/405 ratio	average values/root
0.865	
0.856	
0.883	
0.84	
	0.861
0.71	
0.742	
0.796	
0.801	
	0.76225
0.671	
0.617	
0.589	
0.61	
	0.62175
1.038	
0.903	
0.807	
0.893	
	0.91025
0.661	
0.649	
0.628	
0.721	
	0.66475
0.916	
1.021	
0.98	
1.166	
	1.02075
0.519	
0.528	
0.689	
0.752	
	0.622
0.659	
0.777	
0.759	
0.605	
	0.7
0.716	
0.713	
0.627	
0.704	
	0.69
1.001	
1.113	
1.179	
1.407	
	1.175
0.83	
0.89	
0.816	
0.871	
	0.85175

1h DMSO

458/405 ratio	average values/root
1.3	
1.425	
1.282	
1.363	
	1.3425
0.863	
0.929	
0.82	
0.933	
	0.88625
1.508	
1.706	
1.735	
1.74	
	1.67225
1.054	
1.238	
1.305	
1.101	
	1.1745
1.245	
1.193	
1.147	
1.074	
	1.16475
0.883	
1.064	
1.4	
1.282	
	1.15725
0.833	
1.027	
0.986	
0.992	
	0.9595
1.138	
0.955	
0.914	
1.034	
	1.01025

1h FC

458/405 ratio	average values/root
0.745	
0.755	
0.826	
0.974	
	0.825
1.034	
0.962	
0.999	
0.985	
	0.995
1.081	
1.044	
1.043	
1.053	
	1.05525
0.85	
0.777	
0.781	
0.781	
	0.79725
0.969	
1.004	
1.093	
1.037	
	1.02575
0.725	
0.8	
0.867	
0.926	
	0.8295
0.772	
0.707	
0.72	
0.8	
	0.74975
0.755	
0.73	
0.859	
0.824	
	0.792

1.5h DMSO

458/405 ratio	average values/root
1.607	
1.385	
1.626	
1.686	
	1.576
1.004	
1.171	
1.272	
1.183	
	1.1575
1.972	
1.843	
1.852	
1.685	
	1.838
1.528	
1.92	
2.433	
2.953	
	2.2085
0.972	
1.182	
1.077	
1.379	
	1.1525
1.086	
1.175	
1.18	
1.377	
	1.2045
2.22	
2.088	
2.223	
2.271	
	2.2005
1.072	
1.072	
1.045	
1.035	
	1.056

1.5h FC

458/405 ratio	average values/root
0.973	
0.963	
1.023	
1.293	
	1.063
1.035	
1.172	
1.153	
1.243	
	1.15075
1.042	
1.307	
1.211	
1.012	
	1.143
0.578	
0.631	
0.645	
0.769	
	0.65575
1.165	
1.205	
1.207	
1.198	
	1.19375
0.479	
0.581	
0.64	
0.575	
	0.56875
1.297	
1.211	
1.056	
0.908	
	1.118
