# Supplementary material for: Extracellular matrix sensing by FERONIA and Leucine‐Rich Repeat Extensins controls vacuolar expansion during cellular elongation in Arabidopsis thaliana
Source: EMBO J. 2019 Mar 8;38(7):e100353. doi: 10.15252/embj.2018100353 (PMC6443208; doi:10.15252/embj.2018100353)
Supplement: Supplementary file 6 — Source Data for Appendix [file EMBJ-38-e100353-s013.zip › Figure_S5_Source_Data.pdf]

Appendix Figure S5A

Col-0

length	width	vac. morph. index
17.806	11.063	196.9878
12.577	9.529	119.8462
12.357	5.811	71.80653
6.384	3.882	24.78269
9.895	7.737	76.55762
10.596	5.291	56.06344
8.077	3.882	31.35491
8.903	6.492	57.79828
8.055	5.711	46.00211
12.851	6.078	78.10838
6.732	5.269	35.47091
8.183	4.331	35.44057
12.263	8.406	103.0828
6.611	3.73	24.65903
9.37	4.64	43.4768
6.693	4.947	33.11027
12.833	10.317	132.3981
7.005	5.846	40.95123
7.506	6.007	45.08854
9.383	4.331	40.63777
10.04	5.247	52.67988
8.001	5.776	46.21378
7.586	4.203	31.88396
6.997	4.947	34.61416
10.495	7.37	77.34815
7.351	4.196	30.8448
10.637	6.411	68.19381
7.005	4.953	34.69577
9.78	5.67	55.4526
11.819	4.411	52.13361
8.059	5.604	45.16264
14.591	5.247	76.55898
10.137	5.816	58.95679
9.736	4.196	40.85226
11.533	2.686	30.97764
7.483	3.232	24.18506
14.434	11.076	159.871
13.937	6.487	90.40932
9.73	4.769	46.40237
10.432	6.465	67.44288
10.14	7.636	77.42904
12.338	8.532	105.2678
9.398	6.972	65.52286
7.943	5.308	42.16144

lrx3

length	width	vac. morph. index
11.783	5.91	69.63753
6.93	5.776	40.02768
6.288	4.43	27.85584
10.208	4.489	45.82371
18.266	13.457	245.8056
14.658	16.834	246.7528
13.217	14.815	195.8099
13.474	7.506	101.1358
9.854	5.308	52.30503
9.196	6.007	55.24037
9.243	6.021	55.6521
10.162	4.953	50.33239
12.251	5.489	67.24574
12.016	11.315	135.961
10.958	6.968	76.35534
9.993	7.005	70.00097
9.398	6.766	63.58687
7.636	5.846	44.64006
8.663	4.665	40.4129
9.854	6.766	66.67216
12.244	6.348	77.72491
11.026	7.677	84.6466
13.846	7.307	101.1727
8.495	7.005	59.50748
14.945	7.925	118.4391
8.938	5.968	53.34198
7.099	4.665	33.11684
7.115	5.547	39.46691
9.685	6.487	62.8266
7.307	6.645	48.55502
8.162	6.007	49.02913
12.016	4.829	58.02526
18.45	12.916	238.3002
16.26	8.491	138.0637
13.145	7.099	93.31636
14.466	7.647	110.6215

lrx4

length	width	vac. morph. index
19.738	16.105	317.8805
18.02	13.463	242.6033
15.625	15.144	236.625
17.314	11.535	199.717
13.697	11.292	154.6665
15.093	7.452	112.473
11.795	9.42	111.1089
11.724	7.017	82.26731
14.466	13.463	194.7558
15.29	12.916	197.4856
15.379	12.736	195.8669
14.898	10.596	157.8592
12.166	9.37	113.9954
13.463	7.929	106.7481
11.795	5.531	65.23815
9.149	5.624	51.45398
14.424	11.812	170.3763
15.903	14.688	233.5833
14.656	7.688	112.6753
17.2	13.351	229.6372
19.277	10.04	193.5411
13.712	6.265	85.90568
10.574	6.288	66.48931
13.712	6.288	86.22106
20.428	14.194	289.955
17.599	12.983	228.4878
18.507	13.937	257.9321
18.012	11.055	199.1227
13.695	10.094	138.2373
12.597	8.861	111.622
12.042	11.2	134.8704
10.365	9.181	95.16107
12.023	9.614	115.5891
12.308	6.628	81.57742
7.402	6.158	45.58152
8.34	7.692	64.15128

lrx5

length	width	vac. morph. index
11.929	6.766	80.71161
6.68	5.108	34.12144
9.032	6.343	57.28998
6.676	3.415	22.79854
9.568	6.558	62.74694
8.077	7.464	60.28673
12.301	8.683	106.8096
10.343	5.335	55.17991
16.402	12.035	197.3981
13.566	6.469	87.75845
10.583	6.804	72.00673
10.443	6.663	69.58171
5.696	5.159	29.38566
6.064	4.615	27.98536
5.811	3.058	17.77004
8.709	4.196	36.54296
13.474	7.722	104.0462
12.066	4.64	55.98624
7.815	6.007	46.94471
11.005	3.799	41.808
13.798	10.615	146.4658
10.637	8.077	85.91505
8.65	6.527	56.45855
7.598	5.198	39.4944
11.545	9.373	108.2113
9.358	7.37	68.96846
7.017	6.398	44.89477
6.026	3.604	21.7177
17.301	14.194	245.5704
21.205	12.939	274.3715
19.515	9.898	193.1595
8.769	9.095	79.75406
10.836	9.854	106.7779
12.254	10.162	124.5251
11.533	9.924	114.4535
10.094	7.323	73.91836

lrx3/4

length	width	vac. morph. index
13.474	16.622	223.9648
14.656	10.823	158.6219
19.235	17.806	342.4984
13.704	9.383	128.5846
14.127	9.019	127.4114
11.812	7.943	93.82272
12.977	11.094	143.9668
13.987	13.748	192.2933
12.468	8.522	106.2523
11.775	6.984	82.2366
7.136	6.126	43.71514
15.625	7.929	123.8906
19.896	10.365	206.222
20.938	13.455	281.7208
16.819	14.434	242.7654
13.457	13.987	188.2231
17.566	13.054	229.3066
20.668	18.022	372.4787
16.105	10.376	167.1055
18.026	8.919	160.7739
16.098	12.974	208.8555
19.475	13.704	266.8854
14.424	10.815	155.9956
12.77	11.292	144.1988
15.154	14.448	218.945
10.942	8.515	93.17113
13.03	8.423	109.7517
9.33	6.804	63.48132
15.167	11.055	167.6712
11.543	11.535	133.1485
12.275	6.68	81.997
11.834	7.943	93.99746

lrx3/4/5

length	width	vac. morph. index
17.101	12.494	213.6599
17.794	13.455	239.4183
12.368	10.222	126.4257
14.577	10.117	147.4755
18.779	16.578	311.3183
17.061	15.647	266.9535
15.859	17.074	270.7766
16.126	17.301	278.9959
16.861	14.488	244.2822
18.149	16.641	302.0175
19.221	10.331	198.5722
18.525	7.961	147.4775
17.341	15.205	263.6699
18.519	11.262	208.561
16.585	13.714	227.4467
13.215	14.904	196.9564
16.82	14.178	238.474
15.394	11.783	181.3875
13.968	13.223	184.6989
14.709	10.037	147.6342
14.208	12.754	181.2088
11.775	12.256	144.3144
12.256	13.508	165.554
11.535	14.658	169.08
12.797	8.409	107.61
9.383	7.452	69.92212
10.365	9.196	95.31654
13.249	9.158	121.3343
18.701	13.03	243.674
12.368	8.173	101.0837
13.005	6.611	85.97606
10.836	6.238	67.59497
22.21	14.991	332.9501
16.55	10.194	168.7107
19.019	12.263	233.23
11.2	7.131	79.8672

Appendix Figure S5B

Col-0		average cell length	*lrx3/4/5*		average cell length
length of 4 cells	60.211	15.05275	length of 4 cells	67.416	16.854
	82.29	20.5725		84.676	21.169
	88.042	22.0105		83.962	20.9905
	75.083	18.77075		70.294	17.5735
	79.16	19.79		78.919	19.72975
	78.922	19.7305		67.407	16.85175
	82.792	20.698		77.241	19.31025
	73.164	18.291			
	78.937	19.73425			

Col-0		average cell length	*lrx3/4/5*		average cell length
length of 4 cells	70.398	17.5995	length of 4 cells	98.51	24.6275
	68.476	17.119		59.843	14.96075
	83.372	20.843		92.582	23.1455
	93.223	23.30575		69.677	17.41925
	86.976	21.744		84.333	21.08325
	70.88	17.72		74.027	18.50675
	101.392	25.348		81.69	20.4225
	82.411	20.60275			

Col-0		average cell length	*lrx3/4/5*		average cell length
length of 4 cells	74.003	18.50075	length of 4 cells	78.332	19.583
	78.825	19.70625		85.306	21.3265
	86.744	21.686		79.537	19.88425
	69.692	17.423		84.814	21.2035
	68.536	17.134		49.495	12.37375
	63.707	15.92675		93.231	23.30775
	81.279	20.31975		85.335	21.33375
				75.925	18.98125

Col-0		average cell length	*lrx3/4/5*		average cell length
length of 4 cells	73.282	18.3205	length of 4 cells	69.679	17.41975
	58.625	14.65625		77.126	19.2815
	73.041	18.26025		76.885	19.22125
	81.93	20.4825		88.439	22.10975
	71.839	17.95975		75.697	18.92425
	50.456	12.614		89.619	22.40475
	64.151	16.03775		74.963	18.74075
	90.82	22.705		82.411	20.60275
	63.434	15.8585		84.573	21.14325

Col-0		average cell length	*lrx3/4/5*		average cell length
length of 4 cells	78.086	19.5215	length of 4 cells	76.645	19.16125
	84.339	21.08475		108.926	27.2315
	89.203	22.30075		87.937	21.98425
	65.352	16.338		62.229	15.55725
	93.223	23.30575		91.542	22.8855
	86.496	21.624		70.64	17.66
	70.64	17.66		76.663	19.16575
	109.321	27.33025		82.653	20.66325
	78.087	19.52175		105.477	26.36925

Appendix Figure S5C and D

Col-0 DMSO		Col-0 EGCG		*lrx3/4/5* DMSO		*lrx3/4/5* EGCG	
absolute	relative	absolute	relative	absolute	relative	absolute	relative
2.48	122.3584	1.01	49.83143	1.954	113.6597	1.418	82.48182
1.912	94.33435	1.002	49.43672	1.85	107.6103	1.288	74.92002
2.169	107.0142	0.981	48.40062	1.38	80.27145	1.169	67.99806
1.957	96.55456	1.05	51.80495	2.125	123.6064	1.31	76.19971
2.456	121.1742	0.997	49.19003	2.009	116.8589	1.267	73.6985
1.723	85.00946	0.775	38.23699	1.264	73.52399	1.204	70.03393
1.811	89.3512	0.959	47.31519	1.821	105.9234	0.852	49.55889
1.867	92.11414	0.891	43.9602	1.779	103.4804	1.108	64.44983
1.999	98.62676	0.801	39.51978	1.611	93.70819	1.306	75.96704
1.811	89.3512	0.951	46.92048	1.325	77.07222	1.445	84.05235
2.103	103.7579	1.03	50.81819	1.633	94.98788	1.231	71.60446
2.034	100.3536	0.888	43.81219	1.879	109.2971	1.262	73.40766

Appendix Figure S5E

Col-0 EGCG	*lrx3*	*lrx4*	*lrx5*	*lrx3/4*	*lrx3/4/5*
58.52462	54.65306	67.29461	62.87258	71.20695	91.55429
53.54979	49.45536	53.3752	64.07359	62.65689	92.26402
41.15966	54.55013	59.28833	55.30626	86.08796	67.84954
53.87832	48.88927	62.16641	67.49645	73.75238	75.23066
52.79887	63.55605	46.93878	57.16781	79.23486	86.44429
44.77344	59.95368	69.91104	49.42133	71.59855	80.05678
52.89274	54.96183	51.49137	82.44896	79.62647	86.1604
57.44517	47.19101	51.90999	64.43389	70.489	77.78566
41.25352	60.15953	66.03872	50.44219	72.05543	74.37899
44.77344	58.20396	58.55573	53.74495	69.70579	83.39248
53.40899	54.6016	72.2135	59.50977	85.95743	87.72179
50.96875	46.98516		51.82334	71.01115	78.49539
65.55849	41.68454				
50.20589					
38.66759					
43.72156					
54.54467					
54.44931					
47.63123					
50.44428					
54.78306					
53.40037					
57.35772					
38.47962					
52.64847					
63.63554					
61.79608					
41.31339					
57.42113					
60.9012					
66.27045					
54.63708					
37.58474					
44.39573					
47.17979					
