# Supplementary material for: Extracellular matrix sensing by FERONIA and Leucine‐Rich Repeat Extensins controls vacuolar expansion during cellular elongation in Arabidopsis thaliana
Source: EMBO J. 2019 Mar 8;38(7):e100353. doi: 10.15252/embj.2018100353 (PMC6443208; doi:10.15252/embj.2018100353)
Supplement: Supplementary file 8 — Source Data for Figure 1 [file EMBJ-38-e100353-s006.pdf]

# Figure 1B and C

| early meristem | | | | late meristem | | | | early elongation | | | | late elongation | | | |
|---|---|---|---|---|---|---|---|---|---|---|---|---|---|---|---|
| cell vol. | vac. vol. | occupancy | cytosol | cell vol. | vac. vol. | occupancy | cytosol | cell vol. | vac. vol. | occupancy | cytosol | cell vol. | vac. vol. | occupancy | cytosol |
| 2039 | 937 | 45.95389897 | 1102 | 2201 | 1077.3 | 48.94593367 | 1123.7 | 5317 | 3041.33 | 57.20011285 | 2275.67 | 10100 | 9136 | 90.45544554 | 964 |
| | | | | 1871 | 848.984 | 45.37594869 | 1022.016 | 5005 | 3115.81 | 62.25394605 | 1889.19 | | | | |
| | | | | | | | | 6617 | 4868 | 73.56808221 | 1749 | | | | |
| 1568 | 373.02 | 23.78954082 | 1194.98 | 1925 | 863.67 | 44.86597403 | 1061.33 | 4553 | 3229.03 | 70.92093125 | 1323.97 | 10400 | 9769 | 93.93269231 | 631 |
| | | | | 2762 | 1150.3 | 41.64735699 | 1611.7 | | | | | | | | |
| 1340 | 466.3 | 34.79850746 | 873.7 | 2340 | 1086.7 | 46.44017094 | 1253.3 | 3536 | 2566 | 72.5678733 | 970 | 24000 | 19900 | 82.91666667 | 4100 |
| 681 | 188.78 | 27.72099853 | 492.22 | 1580 | 675.04 | 42.72405063 | 904.96 | 3087 | 1789.99 | 57.98477486 | 1297.01 | 17000 | 14800 | 87.05882353 | 2200 |
| 856 | 315.26 | 36.82943925 | 540.74 | | | | | 4440 | 2255.025 | 50.78885135 | 2184.975 | | | | |
| 1243 | 367.41 | 29.55832663 | 875.59 | 2274 | 893.3 | 39.28320141 | 1380.7 | 5453 | 3132.388 | 57.44338896 | 2320.612 | 16600 | 15000 | 90.36144578 | 1600 |
| | | | | | | | | 6774 | 4526 | 66.81428993 | 2248 | | | | |
| | | | | 2135 | 863.24 | 40.43278689 | 1271.76 | 6327 | 3934.2 | 62.1811285 | 2392.8 | 24200 | 21900 | 90.49586777 | 2300 |
| 1054 | 333.14 | 31.60721063 | 720.86 | 2687 | 1528 | 56.86639375 | 1159 | 3846 | 2954.0768 | 76.80906916 | 891.9232 | 20600 | 15705.55 | 76.24053398 | 4894.45 |
