# Supplementary material for: Extracellular matrix sensing by FERONIA and Leucine‐Rich Repeat Extensins controls vacuolar expansion during cellular elongation in Arabidopsis thaliana
Source: EMBO J. 2019 Mar 8;38(7):e100353. doi: 10.15252/embj.2018100353 (PMC6443208; doi:10.15252/embj.2018100353)
Supplement: Supplementary file 9 — Source Data for Figure 2 [file EMBJ-38-e100353-s007.pdf]

Figure 2 A

DMSO		vac. morph. index	FC		vac. morph. index
length	7.203		length	16.683	
width	5.392	38.838576	width	15.474	258.152742
	6.148			16.077	
	3.44	21.14912		11.662	187.489974
	7.459			13.867	
	2.813	20.982167		8.241	114.277947
	7.896			8.896	
	3.509	27.707064		3.881	34.525376
	6.121			13.918	
	3.509	21.478589		5.228	72.763304
	6.028			10.246	
	3.067	18.487876		8.537	87.470102
	5.186			15.485	
	3.578	18.555508		6.242	96.65737
	5.466			9.278	
	4.019	21.967854		9.498	88.122444
	9.865			12.14	
	4.099	40.436635		6.835	82.9769
	5.747			16.297	
	4.272	24.551184		10.256	167.142032
	3.416			6.859	
	4.069	13.899704		5.088	34.898592
	5.411			10.137	
	5.036	27.249796		6.151	62.352687
	10.169			12.264	
	7.916	80.497804		5.605	68.73972
	7.315			9.479	
	5.841	42.726915		5.572	52.816988
	7.438			10.097	
	5.859	43.579242		8.249	83.290153
	9.479			8.65	
	6.058	57.423782		8.641	74.74465
	12.296			12.166	
	6.386	78.522256		8.392	102.097072
	10.49			10.67	
	4.975	52.18775		6.834	72.91878
	8.07			11.543	
	5.305	42.81135		7.929	91.524447
	5.883			9.956	
	4.639	27.291237		6.252	62.244912
	11.168			12.183	
	6.542	73.061056		6.016	73.292928
	9.087			9.866	
	5.488	49.869456		6.324	62.392584
	7.816			9.264	
	3.813	29.802408		7.439	68.914896
	8.461			9.569	
	7.66	64.81126		6.895	65.978255

Figure 2 B

DMSO		
cell wall	vacuole	occupancy
1797	909	50.58430718
2049	1196	58.36993655
2433	1073	44.10193177
1879	826	43.95955295
2704	1282	47.4112426
3846	1707	44.38377535
3345	1292	38.62481315
2047	1091	53.29750855
2301	796	34.59365493
3365	1611	47.87518574
2330	1032	44.29184549

FC		
cell wall	vacuole	occupancy
4253	2227	52.36303786
2842	1482	52.14637579
2391	1061	44.3747386
3621	2548	70.36730185
2452	1251	51.01957586
4448	2528	56.83453237
5923	3177	53.63835894
3794	1591	41.93463363
2690	1342	49.88847584
3858	2527	65.5002592
3354	2259	67.35241503

Figure 2 C

pER8::GFP-SAUR19 DMSO			pER8::GFP-SAUR19 est		
		vac. morph. index			vac. morph. index
length	16.55		length	7.912	
width	6.089	100.773	width	5.37	42.48744
	7.822			10.054	
	5.118	40.033		7.642	76.83267
	5.136			11.12	
	2.901	14.89954		7.499	83.38888
	7.295			7.854	
	1.949	14.21796		5.907	46.39358
	7.496			14.865	
	3.117	23.36503		11.818	175.6746
	8.074			14.345	
	4.34	35.04116		9.312	133.5806
	4.1			17.622	
	2.667	10.9347		12.498	220.2398
	6.568			6.878	
	1.89	12.41352		5.065	34.83707
	9.528			23.715	
	5.068	48.2879		14.154	335.6621
	9.522			18.581	
	3.898	37.11676		12.758	237.0564
	8.613			11.661	
	4.165	35.87315		8.861	103.3281
	6.286			15.152	
	3.415	21.46669		13.775	208.7188
	6.201			17.311	
	3.831	23.75603		10.059	174.1313
	6.037			12.588	
	4.368	26.36962		9.525	119.9007
	3.244			12.958	
	3.612	11.71733		14.543	188.4482
	6.265			15.54	
	3.964	24.83446		10.236	159.0674
	9.542			10.685	
	5.944	56.71765		8.682	92.76717
	9.231			12.663	
	4.752	43.86571		13.463	170.482
	5.934			9.488	
	3.502	20.78087		7.391	70.12581
	7.75			7.21	
	3.913	30.32575		6.409	46.20889
	7.649			10.271	
	4.736	36.22566		7.091	72.83166
	3.99			9.764	
	3.415	13.62585		6.343	61.93305
	11.215			8.861	
	3.818	42.81887		6.187	54.82301
	3.653			10.376	
	3.64	13.29692		8.409	87.25178
	12.163			10.283	
	6.527	79.3879		6.895	70.90129
	5.433			9.484	
	3.818	20.74319		9.143	86.71221
	5.171			13.591	
	4.399	22.74723		10.534	143.1676
	6.511			8.427	
	4.184	27.24202		4.976	41.93275
	15.743			14.702	
	10.126	159.4136		12.62	185.5392
	8.987			12.271	
	6.431	57.7954		10.327	126.7226
	7.689			10.402	
	6.008	46.19551		9.644	100.3169
	5.62			8.514	
	4.134	23.23308		6.889	58.65295
	10.678			21.502	
	5.167	55.17323		10.494	225.642
	10.869			12.494	
	5.221	56.74705		11.212	140.0827
	9.895			11.551	
	5.16	51.0582		7.451	86.0665
	5.501			8.283	
	4.368	24.02837		4.637	38.40827
	12.082			9.232	
	8.13	98.22666		3.966	36.61411
	7.905			3.846	
	7.144	56.47332		4.266	16.40704
	10.865			3.34	
	4.787	52.01076		1.786	5.96524
	8.313			3.654	
	4.333	36.02023		2.786	10.18004
	18.17			9.778	
	16.784	304.9653		4.796	46.89529
	16.671			6.687	
	10.934	182.2807		4.843	32.38514
	16.098			6.078	
	8.066	129.8465		3.502	21.28516
	20.294			3.492	
	10.745	218.059		2.667	9.313164
	12.961			18.263	
	8.384	108.665		12.531	228.8537
	13.76			18.361	
	6.915	95.1504		11.643	213.7771
	8.993			14.327	
	5.846	52.57308		7.795	111.679
	6.826			12.211	
	3.77	25.73402		9.312	113.7088
	8.044			16.351	
	6.585	52.96974		14.43	235.9449
	9.224			11.376	
	6.019	55.51926		11.814	134.3961
	8.744			15.07	
	7.077	61.88129		9.296	140.0907
	11.886			11.892	
	6.856	81.49042		8.427	100.2139
	10.283			11.12	
	5.802	59.66197		6.915	76.8948
	12.465			10.615	
	6.044	75.33846		7.431	78.88007
	9.378			8.744	
	8.471	79.44104		10.013	87.55367
	6.843			6.162	
	4.62	31.61466		5.166	31.83289
	9.195				
	4.406	40.51317			
	5.171				
	2.886	14.92351			
	3.935				
	2.893	11.38396			
	4.604				
	2.883	13.27333			

Figure 2 D

DMSO

cell wall	vacuole	occupancy
4329	1525	35.22754
3884	1618	41.65808
5829	2979	51.10654
4602	2416	52.49891
3453	1496	43.32465
3810	1353	35.51181
5027	1967	39.1287
3364	1321	39.26873
5627	2674	47.52088
7625	4284	56.18361
8128	3658	45.00492

est

cell wall	vacuole	occupancy
1870	778	41.60428
4777	3105	64.99895
3098	1833	59.1672
3186	1676	52.60515
3916	2468	63.02349
4869	2526	51.87924
6183	4174	67.50768
2893	1836	63.46353

Figure 2 E

pH 5.7

length	width	vac. morph. index
7.692	5.806	44.65975
5.027	3.243	16.30256
3.851	3.06	11.78406
3.557	2.888	10.27262
5.711	4.106	23.44937
8.265	4.165	34.42373
5.985	3.763	22.52156
6.385	3.264	20.84064
10.745	6.465	69.46643
12.773	5.318	67.92681
7.692	6.796	52.27483
7.168	7.066	50.64909
17.62	13.668	240.8302
9.685	5.547	53.7227
13.904	11.315	157.3238
10.812	5.531	59.80117
10.55	5.731	60.46205
6.536	4.251	27.78454
4.865	5.175	25.17638
7.212	4.72	34.04064
21.971	11.605	254.9735
10.23	6.766	69.21618
7.632	6.348	48.44794
11.533	6.745	77.79009
7.272	5.811	42.25759
6.348	4.411	28.00103
5.335	4.571	24.38629
8.522	8.798	74.97656
9.149	5.421	49.59673
13.269	6.064	80.46322
9.346	4.953	46.29074
6.872	4.787	32.89626
12.035	4.811	57.90039
11.384	9.86	112.2462
7.998	8.746	69.95051
5.175	4.665	24.14138
13.234	9.593	126.9538
9.877	8.491	83.86561
15.205	8.001	121.6552
6.288	5.051	31.76069
10.64	10.376	110.4006
9.383	4.953	46.474
7.722	6.984	53.93045
10.103	5.766	58.2539

pH 4.5

length	width	vac. morph. index
23.07	9.42	217.3194
21.625	8.201	177.3466
21.636	14.418	311.9478
17.599	11.076	194.9265
17.011	11.076	188.4138
14.184	8.257	117.1173
13.865	9.481	131.4541
14.448	9.143	132.0981
15.974	10.793	172.4074
18.337	10.423	191.1266
16.31	13.788	224.8823
15.089	11.948	180.2834
14.674	11.384	167.0488
13.215	9.659	127.6437
8.413	6.083	51.17628
10.823	10.103	109.3448
25.044	8.423	210.9456
18.077	13.829	249.9868
16.977	11.505	195.3204
12.256	13.457	164.929
16.212	11.545	187.1675
12.128	9.78	118.6118
8.996	7.208	64.84317
12.066	6.126	73.91632
11.052	13.935	154.0096
14.231	9.52	135.4791
14.658	11.063	162.1615
13.954	10.574	147.5496
13.215	14.658	193.7055
12.263	14.208	174.2327
13.195	13.269	175.0845
13.532	9.877	133.6556
12.77	11.795	150.6222
15.497	11.795	182.7871
16.34	10.196	166.6026
12.016	9.854	118.4057
13.217	9.006	119.0323
11.094	7.005	77.71347
11.505	10.002	115.073
9.565	5.573	53.30575

Figure 2 F

pH 5.7

cell wall	vacuole	occupancy
2425	859	35.42268
2471	1091	44.15217
2970	1342	45.18519
3349	1298	38.75784
2199	997	45.33879
2811	1141	40.59054
2282	1236	54.16301
5072	1925	37.95347
5302	2593	48.90607
2691	1253	46.56262
3336	1950	58.45324

pH 4.5

cell wall	vacuole	occupancy
3915	2322	59.31034
3639	2044	56.16928
3385	1949	57.57755
3750	2306	61.49333
5033	3640	72.32267
5586	3995	71.51808
4888	3252	66.53028
4243	2862	67.45227
4382	2503	57.12004
3927	2192	55.81869
2713	1638	60.37597
