# Supplementary material for: Extracellular matrix sensing by FERONIA and Leucine‐Rich Repeat Extensins controls vacuolar expansion during cellular elongation in Arabidopsis thaliana
Source: EMBO J. 2019 Mar 8;38(7):e100353. doi: 10.15252/embj.2018100353 (PMC6443208; doi:10.15252/embj.2018100353)
Supplement: Supplementary file 10 — Source Data for Figure 3 [file EMBJ-38-e100353-s008.pdf]

Figure 3 A

	DMSO	vac. morph. index		EGCG	vac. morph. index
length	14.664		length	2.605	
width	9.863	144.631	width	1.699	4.425895
	10.334			3.614	
	9.143	94.48376		2.204	7.965256
	14.466			4.854	
	10.091	145.9764		2.549	12.37285
	9.854			2.862	
	6.252	61.60721		2.078	5.947236
	18.6			5.282	
	6.972	129.6792		2.686	14.18745
	16.578			3.372	
	12.743	211.2535		2.166	7.303752
	12.551			3.753	
	7.961	99.91851		2.209	8.290377
	15.184			4.974	
	8.653	131.3872		2.984	14.84242
	11.931			6.738	
	6.384	76.1675		2.548	17.16842
	7.224			4.769	
	4.805	34.71132		2.235	10.65872
	8.077			6.122	
	4.739	38.2769		2.967	18.16397
	6.064			2.508	
	2.739	16.6093		2.635	6.60858
	8.613			4.784	
	7.506	64.64918		2.295	10.97928
	5.017			2.773	
	4.331	21.72863		1.686	4.675278
	10.823			3.41	
	7.272	78.70486		1.672	5.70152
	7.961			3.848	
	5.046	40.17121		1.612	6.202976
	7.87			16.276	
	6.469	50.91103		5.318	86.55577
	4.805			13.215	
	4.251	20.42606		9.398	124.1946
	5.949			6.201	
	5.437	32.34471		5.051	31.32125
	6.492			6.663	
	4.829	31.34987		4.203	28.00459
	4.331			11.665	
	3.465	15.00692		5.604	65.37066
	7.958			5.547	
	6.469	51.4803		4.085	22.6595
	10.334			4.028	
	6.026	62.27268		4.514	18.18239
	11.734			3.911	
	5.108	59.93727		2.718	10.6301
	8.45			6.453	
	4.835	40.85575		4.165	26.87675
	7.748			5.051	
	5.771	44.71371		4.246	21.44655
	7.688			4.516	
	5.097	39.18574		3.667	16.56017
	4.953			5.238	
	4.196	20.78279		2.369	12.40882
	10.128			4.22	
	5.806	58.80317		2.406	10.15332
	20.205			1.956	
	11.333	228.9833		1.733	3.389748
	20.219			5.773	
	9.067	183.3257		2.215	12.7872
	3.911			1.937	
	3.799	14.85789		1.699	3.290963
	19.515			5.806	
	10.509	205.0831		4.134	24.002
	13.668			4.177	
	5.372	73.4245		2.888	12.06318
	8.183			4.093	
	4.665	38.1737		3.009	12.31584
	7.46			1.578	
	5.588	41.68648		1.281	2.021418
	13.904			9.383	
	5.051	70.2291		5.811	54.52461
	9.757			9.65	
	6.14	59.90798		6.77	65.3305
	4.93			4.733	
	4.93	24.3049		6.343	30.02142
	5.372			8.218	
	3.364	18.07141		9.358	76.90404
	8.211			4.949	
	4.787	39.30606		1.937	9.586213
	9.801			4.015	
	6.702	65.6863		3.232	12.97648
	6.343			2.739	
	4.331	27.47153		2.205	6.039495
	10.509			3.366	
	3.675	38.62058		1.686	5.675076
	18.022			3.622	
	8.413	151.6191		1.72	6.22984
	9.423			3.874	
	6.984	65.81023		1.786	6.918964
	9.383			4.029	
	4.733	44.40974		3.1	12.4899
	11.861			1.802	
	6.252	74.15497		2.014	3.629228

Figure 3 B

DMSO

cell wall	vacuole	occupancy
2817	1122	39.82961
3394	1374	40.48321
4162	2048	49.20711
2227	1069	48.0018
2726	1504	55.17241
2357	1020	43.27535
2083	680	32.64522
4037	1757	43.52242
4201	2270	54.03475
2557	1282	50.13688
2488	1211	48.67363
4329	1858	42.91984
3264	1605	49.17279
2514	1331	52.94352
1530	683	44.64052
2352	1115	47.40646

EGCG

cell wall	vacuole	occupancy
5200	2819	54.21154
3120	1361	43.62179
2642	918	34.7464
2619	1185	45.24628
6617	2638	39.86701
4134	1393	33.69618
3180	1204	37.86164
2971	901	30.32649
2682	718	26.77107
2703	669	24.75028
3479	890	25.58206
4109	1471	35.79946
5190	1809	34.85549
2343	565	24.11438
3060	1712	55.94771

Figure 3 C

Col-0 S

		vac. morph. index
length	9.947	
width	5.142	51.14747
	7.402	
	3.16	23.39032
	5.731	
	3.941	22.58587
	7.925	
	6.078	48.16815
	12.275	
	6.984	85.7286
	7.307	
	4.615	33.72181
	10.726	
	5.275	56.57965
	12.503	
	5.711	71.40463
	10.942	
	5.421	59.31658
	12.994	
	4.953	64.35928
	10.094	
	8.368	84.46659
	9.231	
	5.67	52.33977
	7.797	
	6.021	46.94574
	6.972	
	6.265	43.67958
	10.137	
	5.531	56.06775
	7.144	
	3.999	28.56886
	6.834	
	6.011	41.07917
	7.748	
	4.258	32.99098
	7.822	
	4.559	35.6605
	9.505	
	7.525	71.52513
	16.366	
	10.103	165.3457
	13.697	
	6.536	89.52359
	6.628	
	8.893	58.9428
	10.705	
	8.893	95.19957
	8.183	
	6.492	53.12404
	12.577	
	6.252	78.6314
	10.343	
	7.37	76.22791
	15.236	
	5.786	88.1555

Col-0 M

		vac. morph. index
length	8.173	
width	3.338	27.28147
	6.487	
	5.097	33.06424
	4.565	
	4.085	18.64803
	10.162	
	7.448	75.68658
	6.745	
	5.187	34.98632
	7.987	
	2.686	21.45308
	3.133	
	2.802	8.778666
	3.058	
	2.403	7.348374
	5.068	
	2.149	10.89113
	5.159	
	3.498	18.04618
	4.085	
	3.465	14.15453
	4.077	
	3.911	15.94515
	5.776	
	2.554	14.7519
	4.325	
	2.686	11.61695
	4.805	
	2.739	13.1609
	10.216	
	4.805	49.08788
	4.331	
	3.66	15.85146
	5.097	
	5.051	25.74495
	5.766	
	3.852	22.21063
	5.91	
	3.882	22.94262
	4.787	
	3.039	14.54769
	4.258	
	3.604	15.34583
	7.452	
	4.196	31.26859
	9.804	
	4.258	41.74543
	4.859	
	6.064	29.46498
	7.541	
	6.343	47.83256
	5.846	
	5.806	33.94188
	8.532	
	4.411	37.63465

Figure 3 D

Surface

cell wall	vacuole	occupancy
4294	1808	42.10526
3452	1140	33.02433
2621	958	36.55093
3010	1207	40.09967
5444	2254	41.40338
2228	1008	45.24237
5035	1948	38.68918
4328	2204	50.92421
4741	2468	52.05653
3958	1538	38.85801

Medium

cell wall	vacuole	occupancy
1808	552	30.53097
2528	984	38.92405
3255	1023	31.42857
4194	1368	32.61803
2639	1248	47.29064
2875	872	30.33043
2479	810	32.67447
3034	990	32.63019
4465	1706	38.20829
2302	502	21.80712
6573	1886	28.69314
