# Supplementary material for: Extracellular matrix sensing by FERONIA and Leucine‐Rich Repeat Extensins controls vacuolar expansion during cellular elongation in Arabidopsis thaliana
Source: EMBO J. 2019 Mar 8;38(7):e100353. doi: 10.15252/embj.2018100353 (PMC6443208; doi:10.15252/embj.2018100353)
Supplement: Supplementary file 11 — Source Data for Figure 4 [file EMBJ-38-e100353-s009.pdf]

Figure 4 A

	Col-0	vac. morph. index		*fer-4*	vac. morph. index
length	13.619		length	19.005	
width	9.612	130.9058	width	12.2	231.861
	14.622			14.313	
	10.382	151.8056		8.503	121.7034
	15.384			16.686	
	7.849	120.749		7.53	125.6456
	10.211			15.159	
	6.131	62.60364		13.821	209.5125
	5.364			18.583	
	3.993	21.41845		14.473	268.9518
	4.029			16.338	
	2.526	10.17725		10.256	167.5625
	7.689			16.388	
	6.271	48.21772		13.135	215.2564
	5.267			15.07	
	3.063	16.13282		11.551	174.0736
	4.206			18.756	
	2.283	9.602298		11.267	211.3239
	5.407			16.671	
	3.606	19.49764		10.892	181.5805
	4.326			15.087	
	3.486	15.08044		13.15	198.3941
	4.769			14.448	
	3.492	16.65335		13.138	189.8178
	9.983			13.47	
	3.972	39.65248		9.296	125.2171
	5.427			13.79	
	4.142	22.47863		13.455	185.5445
	6.818			10.911	
	2.624	17.89043		15.727	171.5973
	6.577			15.07	
	3.517	23.13131		10.911	164.4288
	8.423			15.07	
	5.709	48.08691		13.17	198.4719
	12.055			12.914	
	5.698	68.68939		10.115	130.6251
	3.123			14.066	
	2.646	8.263458		10.606	149.184
	5.651			13.898	
	4.447	25.13		5.446	75.68851
	7.998			15.377	
	8.267	66.11947		16.174	248.7076
	11.13			17.908	
	7.722	85.94586		10.745	192.4215
	10.758			15.111	
	4.865	52.33767		11.06	167.1277
	6.343			17.923	
	3.338	21.17293		8.512	152.5606
	10.698			16.03	
	6.528	69.83654		13.55	217.2065
	9.566			18.901	
	6.512	62.29379		11.217	212.0125
	5.768			16.982	
	4.1	23.6488		13.79	234.1818
	6.716			21.786	
	5.46	36.66936		17.299	376.876
	13.995			13.197	
	8.461	118.4117		11.604	153.138
	6.314			10.967	
	4.514	28.5014		12.818	140.575
	5.171			10.013	
	3.742	19.34988		9.112	91.23846
	4.769			10.577	
	3.232	15.41341		11.231	118.7903
	11.4			4.816	
	4.355	49.647		4.9	23.5984
	6.408			12.878	
	4.685	30.02148		9.977	128.4838
	7.688			8.009	
	4.811	36.98697		5.766	46.17989
	7.973			7.055	
	5.372	42.83096		7.305	51.53678
	12.008			11.643	
	5.627	67.56902		10.331	120.2838
	7.77			9.427	
	6.409	49.79793		10.948	103.2068
	5.907			16.338	
	4.656	27.50299		9.616	157.1062
	5.877			11.484	
	4.084	24.00167		8.458	97.13167
	10.49			11.814	
	3.874	40.63826		9.557	112.9064
	9.989			12.498	
	3.394	33.90267		11.537	144.1894
	5.831			8.111	
	3.094	18.04111		10.577	85.79005
	5.866			11.267	
	2.583	15.15188		9.946	112.0616
	9.851			9.312	
	5.827	57.40178		9.801	91.26691
	7.63			3.75	
	3.391	25.87333		4.9	18.375
	5.624			12.51	
	3.331	18.73354		7.113	88.98363
	3.742			12.241	
	3.264	12.21389		7.113	87.07023
	9.52			10.572	
	6.408	61.00416		12.531	132.4777
	6.556			13.382	
	5.32	34.87792		11.551	154.5755
	16.073			12.706	
	8.313	133.6148		7.561	96.07007
	8.977			12.51	
	4.787	42.9729		5.89	73.6839
	6.476			12.178	
	3.34	21.62984		9.021	109.8577
	4.953			14.43	
	3.498	17.32559		12.211	176.2047
	6.503			15.46	
	2.906	18.89772		6.415	99.1759
	4.333			16.338	
	2.215	9.597595		10.934	178.6397
	6.12			12.559	
	3.302	20.20824		10.745	134.9465
	4.442			10.692	
	4.004	17.78577		11.189	119.6328
	3.614			9.427	
	4.155	15.01617		7.702	72.60675
	3.345			14.052	
	2.381	7.964445		9.072	127.4797
	14.012				
	6.383	89.4386			
	5.166				
	6.604	34.11626			
	7.396				
	5.166	38.20774			
	6.12				
	3.858	23.61096			

Figure 4 B

Col-0

cell wall	vacuole	occupancy
2610	940	36.01533
2431	939	38.62608
3931	1779	45.25566
1955	937	47.92839
2579	550	21.3261
3583	1893	52.83282
3575	1780	49.79021
2918	1356	46.47019
2077	884	42.56139
1498	517	34.51268
2465	1182	47.95132
1294	578	44.6677

fer-4

cell wall	vacuole	occupancy
8267	5579	67.48518
3799	1965	51.72414
5129	3297	64.28154
4526	2853	63.03579
4637	2986	64.39508
8526	5271	61.82266
3033	1602	52.81899
2769	1107	39.97833
2666	1428	53.56339
2584	1748	67.64706

Figure 4 C

Col-0 DMSO

		vac. morph. index
length	9.685	
width	9.274	89.81869
	8.437	
	6.693	56.46884
	8.392	
	5.318	44.62866
	6.078	
	4.953	30.10433
	13.463	
	5.286	71.16542
	10.06	
	8.155	82.0393
	10.841	
	4.865	52.74147
	7.355	
	3.123	22.96967
	12.279	
	7.066	86.76341
	9.685	
	4.203	40.70606
	8.423	
	3.852	32.4454
	4.947	
	4.085	20.2085
	6.078	
	4.203	25.54583
	9.906	
	7.144	70.76846
	9.611	
	6.536	62.8175
	10.264	
	5.198	53.35227
	4.331	
	3.338	14.45688
	20.668	
	8.893	183.8005
	15.874	
	13.234	210.0765
	9.993	
	7.525	75.19733
	7.475	
	5.776	43.1756
	9.048	
	3.223	29.1617
	5.604	
	4.028	22.57291
	5.269	
	2.215	11.67084
	8.44	
	2.812	23.73328
	3.347	
	3.572	11.95548
	5.531	
	2.176	12.03546
	7.212	
	3.66	26.39592
	13.321	
	9.158	121.9937
	10.531	
	8.077	85.05889
	13.704	
	5.335	73.11084
	9.538	
	7.544	71.95467
	17.728	
	7.212	127.8543
	16.402	
	8.44	138.4329
	7.421	
	3.941	29.24616
	5.291	
	4.59	24.28569
	10.376	
	8.073	83.76545
	10.137	
	3.911	39.64581
	5.846	
	3.347	19.56656
	7.131	
	3.44	24.53064
	5.816	
	3.415	19.86164
	5.421	
	3.011	16.32263
	3.911	
	2.176	8.510336
	5.624	
	3.799	21.36558
	8.864	
	6.126	54.30086
	9.37	
	8.183	76.67471
	6.558	
	2.279	14.94568
	4.559	
	2.643	12.04944
	15.617	
	6.732	105.1336
	6.884	
	6.021	41.44856
	9.032	
	7.954	71.84053
	5.816	
	6.558	38.14133

Col-0 EGCG

		vac. morph. index
length	8.919	
width	5.291	47.19043
	5.776	
	3.223	18.61605
	6.361	
	3.372	21.44929
	5.711	
	5.846	33.38651
	10.555	
	5.67	59.84685
	8.158	
	5.944	48.49115
	5.588	
	5	27.94
	4.859	
	2.972	14.44095
	9.924	
	2.654	26.3383
	2.903	
	2.599	7.544897
	2.403	
	2.599	6.245397
	2.718	
	2.366	6.430788
	7.826	
	9.246	72.3592
	9.757	
	5.198	50.71689
	6.007	
	3.268	19.63088
	6.247	
	3.133	19.57185
	14.569	
	5.136	74.82638
	8.368	
	4.203	35.1707
	5.051	
	3.604	18.2038
	7.954	
	3.206	25.50052
	15.444	
	10.14	156.6022
	10.025	
	5.335	53.48338
	3.852	
	2.893	11.14384
	6.536	
	4.469	29.20938
	6.796	
	4.106	27.90438
	7.748	
	2.654	20.56319
	6.745	
	3.347	22.57552
	3.133	
	2.893	9.063769
	7.2	
	2.149	15.4728
	5.437	
	3.268	17.76812
	5.479	
	2.739	15.00698
	4.947	
	2.403	11.88764
	5.175	
	2.686	13.90005
	4.331	
	1.733	7.505623
	4.331	
	3.372	14.60413
	6.007	
	2.599	15.61219
	3.604	
	3.999	14.4124
	4.203	
	3.799	15.9672
	4.417	
	2.812	12.4206
	3.604	
	3.133	11.29133
	4.251	
	3.338	14.18984
	3.572	
	3.268	11.6733
	4.953	
	3.268	16.1864
	3.874	
	2.739	10.61089
	3.232	
	3.572	11.5447
	5.806	
	5.091	29.55835
	7.903	
	4.469	35.31851
	2.415	
	2.903	7.010745
	3.667	
	2.588	9.490196
	2.893	
	2.279	6.593147
	2.366	
	3.133	7.412678
	5.91	
	3.133	18.51603

***fer-4* DMSO**

		vac. morph. index
length	16.354	
width	13.217	216.1508
	20.445	
	14.208	290.4826
	18.514	
	12.503	231.4805
	18.987	
	13.695	260.027
	12.994	
	9.209	119.6617
	11.573	
	7.07	81.82111
	11.665	
	7.355	85.79608
	12.403	
	5.489	68.08007
	16.345	
	10.103	165.1335
	16.242	
	9.143	148.5006
	14.249	
	11.333	161.4839
	13.448	
	7.856	105.6475
	21.448	
	12.842	275.4352
	21.405	
	16.55	354.2528
	17.074	
	14.424	246.2754
	18.149	
	14.249	258.6051
	15.689	
	12.042	188.9269
	14.664	
	11.107	162.873
	19.68	
	14.937	293.9602
	10.216	
	7.475	76.3646
	11.076	
	12.503	138.4832
	19.467	
	7.987	155.4829
	14.834	
	8.211	121.802
	15.619	
	6.972	108.8957
	16.764	
	11.2	187.7568
	12.641	
	11.397	144.0695
	14.896	
	8.413	125.32
	13.987	
	9.863	137.9538
	16.752	
	12.551	210.2544
	17.465	
	13.269	231.7431
	20.445	
	13.532	276.6617
	14.658	
	12.338	180.8504
	23.085	
	14.896	343.8742
	23.551	
	15.139	356.5386
	16.88	
	12.016	202.8301
	20.258	
	10.596	214.6538
	12.815	
	9.209	118.0133
	11.622	
	5.655	65.72241
	11.812	
	6.597	77.92376
	14.664	
	7.212	105.7568
	19.04	
	11.455	218.1032
	18.894	
	11.356	214.5603
	22.998	
	12.93	297.3641
	19.978	
	15.379	307.2417

***fer-4* EGCG**

		vac. morph. index
length	21.385	
width	11.543	246.8471
	14.34	
	8.495	121.8183
	18.213	
	8.218	149.6744
	13.269	
	12.073	160.1966
	25.269	
	16.338	412.8449
	23.984	
	15.593	373.9825
	15.129	
	12.93	195.618
	20.659	
	12.251	253.0934
	12.272	
	5.187	63.65486
	18.034	
	6.972	125.733
	11.692	
	11.262	131.6753
	11.834	
	8.65	102.3641
	13.457	
	16.345	219.9547
	11.535	
	15.047	173.5671
	14.954	
	14.442	215.9657
	24.989	
	11.535	288.2481
	17.326	
	12.275	212.6767
	12.736	
	8.413	107.148
	19.078	
	13.01	248.2048
	15.424	
	16.185	249.6374
	12.905	
	11.177	144.2392
	17.492	
	11.753	205.5835
	10.572	
	6.265	66.23358
	13.455	
	9.37	126.0734
	21.177	
	12.77	270.4303
	11.573	
	10.244	118.5538
	18.419	
	12.431	228.9666
	11.714	
	7.228	84.66879
	12.977	
	10.343	134.2211
	11.535	
	13.489	155.5956
	15.23	
	11.893	181.1304
	15.351	
	7.136	109.5447
	18.955	
	15.332	290.6181
	15.858	
	10.815	171.5043
	14.966	
	14.151	211.7839
	16.481	
	10.878	179.2803
	20.059	
	13.697	274.7481
	16.126	
	10.642	171.6129
	15.634	
	7.351	114.9255
	15.728	
	10.67	167.8178
	18.557	
	9.383	174.1203
	14.418	
	8.423	121.4428
	26.07	
	17.366	452.7316
	24.789	
	13.457	333.5856

Figure 4 D

	Col-0 S	vac. morph. index		Col-0 M	vac. morph. index		*fer-4* S	vac. morph. index		*fer-4* M	vac. morph. index
length	15.06		length	6.126		length	12.655		length	11.262	
width	7.822	117.7993	width	4.645	28.45527	width	12.013	152.0245	width	7.402	83.36132
	11.303			5.467			11.661			8.942	
	5.308	59.99632		3.405	18.61514		5.804	67.68044		8.532	76.29314
	8.514			3.06			11.274			12.251	
	6.932	59.01905		2.666	8.15796		8.246	92.9654		8.706	106.6572
	13.01			3.405			10.737			9.181	
	5.846	76.05646		2.607	8.876835		10.413	111.8044		7.943	72.92468
	12.329			5.166			12.577			18.725	
	10.014	123.4626		3.333	17.21828		13.153	165.4253		13.109	245.466
	9.685			4.67			21.143			17.546	
	5.816	56.32796		4.165	19.45055		12.166	257.2257		8.942	156.8963
	7.212			10.567			18.274			14.145	
	5.573	40.19248		7.615	80.46771		12.743	232.8656		10.395	147.0373
	12.403			5.724			12.974			12.308	
	5.766	71.5157		5.217	29.86211		13.695	177.6789		5.604	68.97403
	13.217			6.095			12.035			12.251	
	8.663	114.4989		3.398	20.71081		9.196	110.6739		9.78	119.8148
	14.543			4.553			16.957			14.324	
	6.527	94.92216		3.398	15.47109		10.583	179.4559		9.593	137.4101
	14.448			3.159			16.827			11.731	
	11.356	164.0715		2.728	8.617752		15.351	258.3113		7.87	92.32297
	7.475			4.779			14.688			14.434	
	7.596	56.7801		4.762	22.7576		14.249	209.2893		10.836	156.4068
	16.58			6.395			14.466			16.594	
	5.291	87.72478		4.762	30.45299		13.153	190.2713		13.489	223.8365
	10.596			5.128			19.132			19.414	
	3.604	38.18798		4.553	23.34778		14.231	272.2675		10.395	201.8085
	8.503			5.831			14.752			17.541	
	3.528	29.99858		3.398	19.81374		14.208	209.5964		12.641	221.7358
	6.88			5.111			13.112			15.796	
	4.093	28.15984		4.313	22.04374		6.384	83.70701		9.281	146.6027
	10.808			7.611			13.76			17.101	
	7.055	76.25044		3.528	26.85161		12.916	177.7242		18.046	308.6046
	10.196			6.536			14.543			15.865	
	4.903	49.99099		2.728	17.83021		12.099	175.9558		16.585	263.121
	8.023			7.238			15.974			14.037	
	5.615	45.04915		3.379	24.4572		13.249	211.6395		17.406	244.328
	6.255			6.099			16.114			12.403	
	4.43	27.70965		4.229	25.79267		14.178	228.4643		8.001	99.2364
	8.68			9.932			12.734			22.346	
	6.201	53.82468		7.224	71.74877		12.994	165.4656		15.377	343.6144
	8.663			4.017			15.619			21.384	
	4.622	40.04039		3.498	14.05147		12.974	202.6409		7.452	159.3536
	7.406			5.247			14.434			15.354	
	4.232	31.34219		3.513	18.43271		14.208	205.0783		9.804	150.5306
	7.598			5.474			14.488			11.023	
	5.091	38.68142		3.614	19.78304		10.596	153.5148		8.919	98.31414
	14.219			9.006			17.541			19.998	
	5.742	81.6455		4.872	43.87723		16.354	286.8655		13.63	272.5727
	8.608			10.283			18.022			16.788	
	6.055	52.12144		5.946	61.14272		15.619	281.4856		9.505	159.5699
	10.649			11.081			14.448			15.205	
	6.347	67.5892		6.637	73.5446		15.684	226.6024		10.855	165.0503
	12.03			4.204			12.013			15.424	
	5.308	63.85524		4.229	17.77872		9.174	110.2073		9.623	148.4252
