# Supplementary material for: Extracellular matrix sensing by FERONIA and Leucine‐Rich Repeat Extensins controls vacuolar expansion during cellular elongation in Arabidopsis thaliana
Source: EMBO J. 2019 Mar 8;38(7):e100353. doi: 10.15252/embj.2018100353 (PMC6443208; doi:10.15252/embj.2018100353)
Supplement: Supplementary file 12 — Source Data for Figure 5 [file EMBJ-38-e100353-s010.pdf]

Figure 5 A

Col-0

length	width	vac. morph. index
9.623	9.143	87.98309
11.515	4.835	55.67503
9.863	4.805	47.39172
11.731	5.437	63.78145
10.726	4.622	49.57557
9.456	6.201	58.63666
7.826	4.258	33.32311
3.268	4.769	15.58509
19.627	11.897	233.5024
16.105	7.929	127.6965
13.269	6.252	82.95779
12.18	4.489	54.67602
8.812	4.559	40.17391
8.821	4.331	38.20375
9.143	4.665	42.6521
6.288	4.092	25.7305
10.025	8.706	87.27765
5.934	4.953	29.3911
4.865	4.411	21.45952
5.051	3.415	17.24917
8.03	4.411	35.42033
7.987	7.987	63.79217
8.501	3.753	31.90425
4.622	3.268	15.1047
12.401	7.932	98.36473
7.483	7.987	59.76672
16.276	7.402	120.475
7.351	4.953	36.4095
10.062	9.133	91.89625
10.264	8.586	88.1267
9.149	5.609	51.31674
5.776	4.251	24.55378
8.001	8.893	71.15289
11.455	9.243	105.8786
12.503	6.984	87.32095
8.746	5.247	45.89026
6.201	2.933	18.18753
3.133	3.077	9.640241
7.688	3.133	24.0865
4.622	4.59	21.21498
9.358	7.095	66.39501
9.019	4.93	44.46367
3.268	2.053	6.709204
6.505	5.624	36.58412
15.053	9.697	145.9689
10.401	7.212	75.01201
11.063	5.547	61.36646
8.903	8.183	72.85325
10.137	8.703	88.22231
8.663	5.372	46.53764
6.126	3.415	20.92029
7.903	4.085	32.28376

lrx3/4/5

length	width	vac. morph. index
11.692	13.948	163.08
11.31	11.11	125.6541
13.476	9.906	133.4933
11.315	6.288	71.14872
22.346	13.704	306.2296
18.816	8.626	162.3068
14.226	15.205	216.3063
10.823	8.919	96.53034
22.086	13.628	300.988
21.149	14.176	299.8082
14.514	12.606	182.9635
11.356	13.234	150.2853
14.096	10.196	143.7228
13.116	9.757	127.9728
11.783	7.845	92.43764
5.447	5.816	31.67975
17.832	9.906	176.6438
13.888	12.734	176.8498
6.411	5.051	32.38196
6.732	6.154	41.42873
23.791	12.977	308.7358
20.458	12.754	260.9213
13.215	9.481	125.2914
15.903	8.68	138.038
19.019	11.861	225.5844
19.392	12.916	250.4671
15.881	13.003	206.5006
12.939	10.222	132.2625
17.449	10.921	190.5605
12.023	11.893	142.9895
8.556	5.786	49.50502
10.105	4.835	48.85768
13.944	11.063	154.2625
13.532	9.623	130.2184
10.64	7.335	78.0444
5.731	4.533	25.97862
13.489	9.133	123.195
13.987	8.68	121.4072
11.2	7.666	85.8592
11.773	7.929	93.34812
19.176	15.129	290.1137
18.982	13.249	251.4925
15.887	14.418	229.0588
13.249	13.695	181.4451
21.165	12.494	264.4355
19.235	14.664	282.062
21.396	14.061	300.8492
15.706	8.33	130.831

Figure 5 B

Col-0

cell wall	vacuole	occupancy
5243	1931	36.83006
4335	1410	32.52595
3277	1128	34.42173
4183	2597	62.08463
2153	1533	71.20297
2165	1020	47.11316
3487	1708	48.98193
4262	1745	40.94322
2956	1469	49.69553
4591	2401	52.29797
6220	3048	49.00322

lrx3/4/5

cell wall	vacuole	occupancy
3729	2206	59.15795
4243	2612	61.56022
4178	3275	78.38679
3967	2971	74.89287
4124	2902	70.36857
4334	2143	49.44624
6380	4463	69.95298
5390	3113	57.7551
5163	3984	77.16444
3301	2300	69.67586

Figure 5 C

	Col-0 DMSO	vac. morph. index
length	9.243	
width	5.811	53.71107
	11.094	
	6.158	68.31685
	10.137	
	6.007	60.89296
	11.026	
	7.07	77.95382
	10.823	
	4.665	50.4893
	7.034	
	6.558	46.12897
	5.89	
	3.372	19.86108
	7.452	
	3.465	25.82118
	7.506	
	3.738	28.05743
	9.149	
	4.835	44.23542
	7.46	
	4.298	32.06308
	6.469	
	3.572	23.10727
	14.01	
	9.665	135.4067
	10.284	
	7.666	78.83714
	6.934	
	6.384	44.26666
	6.645	
	3.667	24.36722
	8.938	
	5.437	48.59591
	11.177	
	8.919	99.68766
	7.521	
	3.077	23.14212
	5.609	
	5.291	29.67722
	16.162	
	10.334	167.0181
	12.613	
	8.211	103.5653
	9.617	
	4.196	40.35293
	16.622	
	6.265	104.1368
	11.736	
	6.126	71.89474
	10.705	
	4.384	46.93072
	9.898	
	6.505	64.38649
	5.604	
	5.696	31.92038
	9.886	
	6.872	67.93659
	7.632	
	3.667	27.98654
	9.505	
	4.417	41.98359
	4.113	
	2.739	11.26551
	7.598	
	5.291	40.20102
	6.838	
	4.331	29.61538
	9.851	
	2.812	27.70101
	7.212	
	4.469	32.23043
	10.401	
	6.361	66.16076
	15.881	
	6.597	104.767
	8.34	
	5.335	44.4939
	5.247	
	4.43	23.24421

	lrx3/4/5 DMSO	vac. morph. index
length	14.914	
width	12.099	180.4445
	14.727	
	12.338	181.7017
	16.846	
	12.754	214.8539
	16.354	
	17.539	286.8328
	11.558	
	5.711	66.00774
	8.653	
	5.068	43.8534
	11.553	
	6.88	79.48464
	8.938	
	8.841	79.02086
	13.937	
	12.515	174.4216
	10.981	
	6.834	75.04415
	10.222	
	6.68	68.28296
	16.82	
	11.543	194.1533
	15.384	
	11.832	182.0235
	11.929	
	9.33	111.2976
	12.645	
	12.251	154.9139
	13.532	
	14.145	191.4101
	12.487	
	9.736	121.5734
	16.57	
	10.637	176.2551
	16.013	
	9.465	151.563
	13.995	
	12.016	168.1639
	14.231	
	7.903	112.4676
	8.491	
	6.834	58.02749
	8.358	
	11.117	92.91589
	11.11	
	9.993	111.0222
	16.34	
	11.333	185.1812
	22.715	
	12.475	283.3696
	14.102	
	9.398	132.5306
	14.898	
	10.467	155.9374
	12.736	
	11.861	151.0617
	17.838	
	12.263	218.7474
	14.898	
	8.653	128.9124
	11.384	
	6.126	69.73838
	17.061	
	14.424	246.0879
	11.795	
	15.384	181.4543
	15.139	
	13.714	207.6162
	13.798	
	12.256	169.1083

	Col-0 EGCG	vac. morph. index
length	4.947	
width	3.73	18.45231
	3.799	
	3.977	15.10862
	2.893	
	2.267	6.558431
	2.739	
	2.739	7.502121
	3.941	
	2.067	8.146047
	4.865	
	2.599	12.64414
	4.565	
	2.176	9.93344
	1.749	
	3.039	5.315211
	8.746	
	4.028	35.22889
	6.288	
	4.148	26.08262
	3.753	
	4.092	15.35728
	5.655	
	5.187	29.33249
	4.533	
	3.911	17.72856
	7.307	
	4.769	34.84708
	3.039	
	2.739	8.323821
	5.786	
	3.338	19.31367
	6.884	
	3.415	23.50886
	6.766	
	2.802	18.95833
	5.484	
	3.667	20.10983
	2.474	
	2.718	6.724332
	7.37	
	5.136	37.85232
	3.874	
	2.883	11.16874
	8.073	
	3.465	27.97295
	5.372	
	3.465	18.61398
	4.665	
	4.077	19.01921
	4.077	
	3.604	14.69351
	3.039	
	2.883	8.761437
	3.799	
	2.508	9.527892
	6.68	
	5.484	36.63312
	4.533	
	5.108	23.15456
	5.269	
	2.933	15.45398
	5.944	
	3.347	19.89457
	9.529	
	5.447	51.90446
	4.148	
	3.123	12.9542
	8.073	
	4.251	34.31832
	6.177	
	3.882	23.97911
	8.812	
	5.609	49.42651
	5.175	
	6.307	32.63873
	6.288	
	3.415	21.47352
	4.72	
	3.66	17.2752
	10.002	
	7.005	70.06401
	7.598	
	4.93	37.45814
	6.233	
	2.893	18.03207
	5.949	
	3.66	21.77334

	lrx3/4/5 EGCG	vac. morph. index
length	10.365	
width	7.506	77.79969
	10.002	
	5.269	52.70054
	7.958	
	5.399	42.96524
	8.812	
	3.882	34.20818
	18.804	
	12.368	232.5679
	17.643	
	11.714	206.6701
	10.615	
	8.409	89.26154
	9.538	
	7.692	73.3663
	16.114	
	10.583	170.5345
	16.345	
	8.919	145.7811
	17.306	
	12.291	212.708
	17.789	
	11.485	204.3067
	17.806	
	14.416	256.6913
	16.923	
	11.897	201.3329
	16.834	
	9.565	161.0172
	18.262	
	11.535	210.6522
	16.117	
	13.116	211.3906
	17.877	
	13.813	246.935
	15.81	
	11.213	177.2775
	10.921	
	6.307	68.87875
	11.13	
	6.252	69.58476
	13.935	
	14.658	204.2592
	18.26	
	12.736	232.5594
	10.222	
	7.296	74.57971
	17.262	
	10.769	185.8945
	21.625	
	15.377	332.5276
	11.832	
	8.663	102.5006
	17.786	
	10.836	192.7291
	15.617	
	8.586	134.0876
	9.057	
	8.105	73.40699
	7.737	
	7.475	57.83408
	12.878	
	9.149	117.8208
	11.333	
	7.782	88.19341
	17.074	
	14.208	242.5874
	19.725	
	13.954	275.2427
	18.502	
	13.354	247.0757

Figure 5 D

S
Col-0

		vac. morph. index
length	7.597	
width	4.749	36.07815
	7.679	
	3.868	29.70237
	8.991	
	7.862	70.68724
	7.358	
	3.841	28.26208
	8.97	
	4.893	43.89021
	7.678	
	4.882	37.484
	9.87	
	6.446	63.62202
	12.154	
	7.044	85.61278
	15.829	
	9.371	148.3336
	10.747	
	4.771	51.27394
	9.788	
	5.117	50.0852
	10.725	
	9.616	103.1316
	10.614	
	7.577	80.42228
	7.862	
	5.299	41.66074
	12.158	
	6.574	79.92669
	6.405	
	6.077	38.92319
	8.968	
	10.7	95.9576
	10.917	
	6.559	71.6046
	10.324	
	4.291	44.30028
	8.832	
	5.28	46.63296
	9.457	
	4.808	45.46926
	11.605	
	4.549	52.79115
	6.054	
	5.496	33.27278
	8.299	
	4.515	37.46999
	12.973	
	11.517	149.41
	16.184	
	11.697	189.3042
	8.692	
	6.255	54.36846
	11.026	
	9.118	100.5351
	13.738	
	9.67	132.8465
	9.892	
	8.086	79.98671
	6.035	
	5.586	33.71151
	5.658	
	5.246	29.68187
	12.334	
	8.882	109.5506
	7.803	
	5.428	42.35468
	9.412	
	4.84	45.55408
	7.644	
	4.771	36.46952
	8.97	
	4.893	43.89021
	10.075	
	5.759	58.02193
	7.132	
	3.297	23.5142
	9.025	
	5.634	50.84685

M
Col-0

		vac. morph. index
length	4.633	
width	3.198	14.81633
	6.734	
	6.564	44.20198
	5.821	
	3.791	22.06741
	6.035	
	3.747	22.61315
	10.676	
	4.657	49.71813
	8.797	
	4.64	40.81808
	6.369	
	3.904	24.86458
	7.203	
	2.918	21.01835
	5.795	
	3.551	20.57805
	5.28	
	3.043	16.06704
	3.92	
	3.005	11.7796
	5.507	
	2.936	16.16855
	12.187	
	9.118	111.1211
	10.123	
	5.841	59.12844
	14.632	
	8.399	122.8942
	11.803	
	8.149	96.18265
	4.874	
	3.626	17.67312
	4.549	
	3.533	16.07162
	4.455	
	3.445	15.34748
	4.649	
	4.391	20.41376
	8.512	
	5.766	49.08019
	7.069	
	6.269	44.31556
	6.718	
	5.936	39.87805
	5.586	
	3.933	21.96974
	7.184	
	4.811	34.56222
	7.125	
	2.936	20.919
	6.943	
	3.551	24.65459
	7.393	
	5.768	42.64282
	11.806	
	4.615	54.48469
	4.926	
	4.662	22.96501
	7.757	
	5.821	45.1535
	5.458	
	3.92	21.39536
	11.835	
	7.691	91.02299
	5.971	
	3.392	20.25363
	4.604	
	4.225	19.4519
	5.1	
	3.682	18.7782
	7.372	
	5.871	43.28101
	9.154	
	5.654	51.75672
	4.808	
	2.245	10.79396
	4.582	
	2.797	12.81585
	16.319	
	11.761	191.9278
	17.166	
	6.994	120.059
	12.593	
	6.6	83.1138
	6.144	
	5.391	33.1223
	6.241	
	8.212	51.25109
	12.467	
	5.364	66.87299
	6.793	
	4.085	27.74941
	5.266	
	4.127	21.73278

S
lrx3/4/5

		vac. morph. index
length	14.411	
width	13.467	194.0729
	17.031	
	11.517	196.146
	8.282	
	5.615	46.50343
	12.556	
	9.367	117.6121
	17.7	
	8.518	150.7686
	19.467	
	11.761	228.9514
	14.593	
	11.277	164.5653
	15.028	
	12.588	189.1725
	15.181	
	10.34	156.9715
	14.591	
	5.299	77.31771
	16.793	
	12.348	207.36
	8.875	
	4.779	42.41363
	19.443	
	12.962	252.0202
	16.216	
	12.989	210.6296
	16.073	
	12.962	208.3382
	8.927	
	16.819	150.1432
	8.695	
	6.459	56.16101
	13.707	
	8.488	116.345
	18.988	
	12.439	236.1917
	21.467	
	11.994	257.4752
	16.424	
	12.032	197.6136
	18.237	
	11.037	201.2818
	18.526	
	11.524	213.4936
	15.16	
	10.794	163.637
	14.88	
	8.187	121.8226
	18.658	
	11.161	208.2419
	15.621	
	9.847	153.82
	11.045	
	6.547	72.31162

M
lrx3/4/5

		vac. morph. index
length	19.671	
width	11.037	217.1088
	14.155	
	12.51	177.0791
	16.159	
	11.315	182.8391
	12.824	
	12.054	154.5805
	16.853	
	15.594	262.8057
	22.132	
	15.861	351.0357
	19.443	
	14.171	275.5268
	19.915	
	16.287	324.3556
	15.36	
	11.517	176.9011
	12.365	
	8.562	105.8691
	11.378	
	10.94	124.4753
	8.649	
	7.71	66.68379
	16.2	
	11.697	189.4914
	11.756	
	9.835	115.6203
	11.579	
	11.994	138.8785
	12.139	
	10.18	123.575
	7.087	
	5.538	39.24781
	12.75	
	6.785	86.50875
	9.144	
	6.721	61.45682
	6.755	
	5.128	34.63964
	13.271	
	13.987	185.6215
	8.45	
	7.89	66.6705
	15.205	
	9.166	139.369
	7.81	
	6.073	47.43013
	17.517	
	11.996	210.1339
	18.12	
	14.634	265.1681
	9.343	
	9.462	88.40347
	11.697	
	8.914	104.2671
	14.65	
	10.317	151.1441
	13.946	
	8.45	117.8437
	16.853	
	10.579	178.2879
	8.732	
	7.216	63.01011
