# Supplementary material for: Extracellular matrix sensing by FERONIA and Leucine‐Rich Repeat Extensins controls vacuolar expansion during cellular elongation in Arabidopsis thaliana
Source: EMBO J. 2019 Mar 8;38(7):e100353. doi: 10.15252/embj.2018100353 (PMC6443208; doi:10.15252/embj.2018100353)

Figure 6 B

HA beads pulldown and Anti-FLAG antibody detection

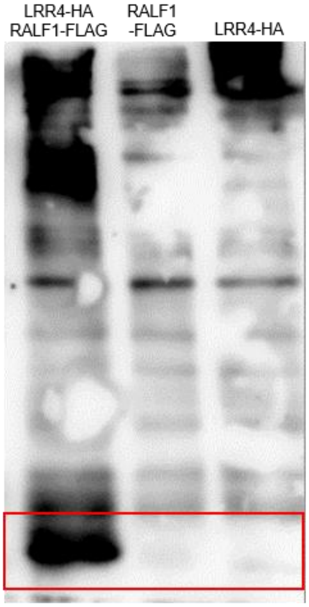

FLAG beads pulldown and Anti-HA antibody detection

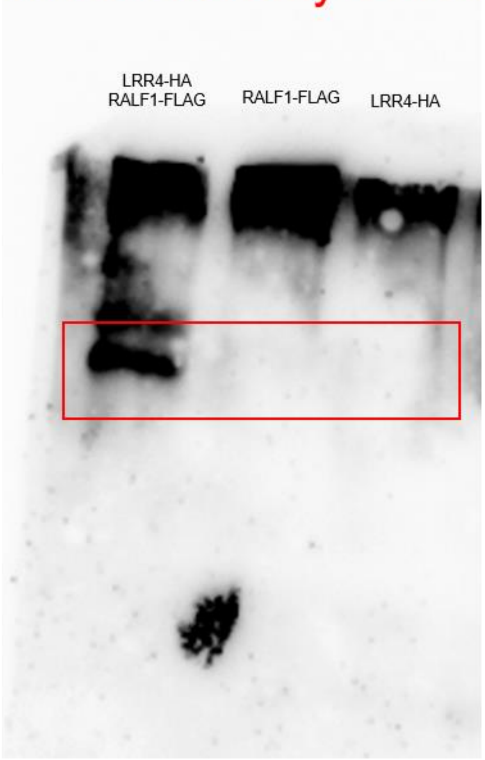

Anti-HA total protein extract detection

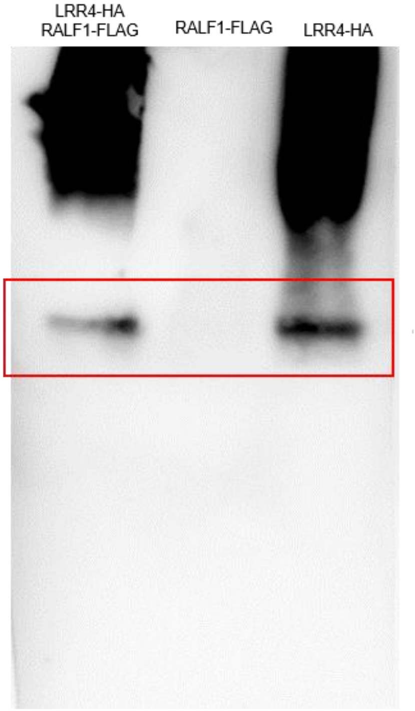

Anti-FLAG total protein extract detection

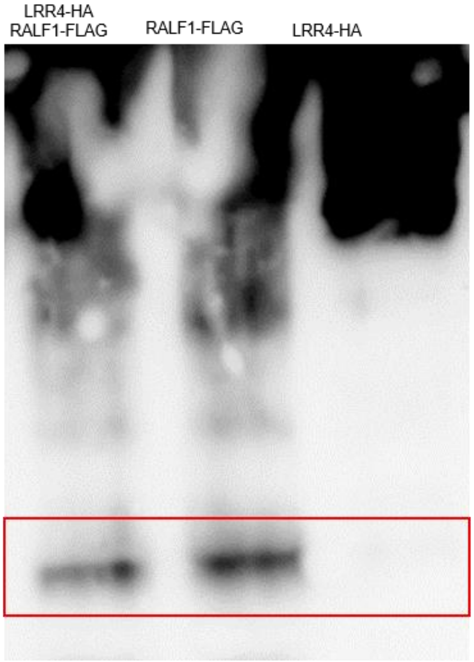

Figure 6 C

Col-0

control		1 µM		1.25 µM		1.5 µM	
absolute	relative	absolute	relative	absolute	relative	absolute	relative
1.983	91.85195	1.063	49.23783	1.045	48.40408	0.752	34.83241
2.251	104.2656	1.211	56.09314	1.034	47.89456	0.717	33.21122
2.171	100.56	1.24	57.43642	1.043	48.31144	0.695	32.19218
2.415	111.8621	1.16	53.73084	0.866	40.11285	0.689	31.91427
2.1	97.27135	1.074	49.74735	1.272	58.91865	0.687	31.82163
1.992	92.26882	1.073	49.70103	0.977	45.25434	0.767	35.5272
2.201	101.9496	1.274	59.01129	1.044	48.35776	0.671	31.08051
2.22	102.8297	1.217	56.37106	0.969	44.88378	0.657	30.43204
2.292	106.1647	1.317	61.00303	1.206	55.86155	0.703	32.56274
2.163	100.1895	1.074	49.74735			0.543	25.15159
1.96	90.78659	1.354	62.71686			0.615	28.48661
		1.247	57.76065				

fer-4

control		1 µM		1.25 µM		1.5 µM	
absolute	relative	absolute	relative	absolute	relative	absolute	relative
1.207	83.51496	1.206	83.44577	1.756	121.5015	1.487	102.8888
1.325	91.67964	1.334	92.30237	1.575	108.9777	1.153	79.77859
1.202	83.169	1.193	82.54627	1.345	93.06348	1.225	84.76042
1.266	87.5973	1.122	77.63363	1.466	101.4357	1.238	85.65992
1.484	102.6812	1.875	129.7353	1.438	99.49836	1.679	116.1737
1.576	109.0469	1.249	86.42103	1.298	89.81145	1.184	81.92354
1.275	88.22003	1.616	111.8146	1.143	79.08666	1.382	95.62359
1.837	127.106	1.471	101.7817	1.038	71.82148	1.615	111.7454
1.603	110.9151	1.426	98.66805	1.393	96.38471	1.156	79.98616
1.309	90.57257	1.223	84.62204	1.81	125.2378	1.045	72.30583
1.278	88.42761	1.132	78.32555	1.31	90.64176	1.271	87.94326
1.981	137.0697						

lrx3/4/5

control		1 µM		1.25 µM		1.5 µM	
absolute	relative	absolute	relative	absolute	relative	absolute	relative
1.759	97.83511	1.133	63.01716	1.004	55.84221	0.889	49.44594
2.014	112.0181	1.051	58.45634	1.082	60.18055	0.749	41.65918
1.916	106.5674	0.971	54.00676	0.832	46.27562	0.661	36.76464
1.748	97.22329	1.188	66.07624	1.045	58.12262	0.728	40.49117
1.719	95.61032	0.981	54.56296	1.048	58.28948	0.649	36.09721
1.686	93.77487	0.929	51.67073	0.987	54.89668	0.72	40.04621
1.898	105.5663	0.987	54.89668	0.802	44.60703	0.797	44.32893
1.611	89.60339	1.322	73.52929	0.762	42.38224	0.721	40.10183
1.862	103.5639	1.171	65.13071	1.083	60.23617	0.676	37.59894
1.461	81.26043			0.894	49.72404	0.775	43.10529
1.833	101.951			0.928	51.61511	0.846	47.05429
2.16	120.1386			0.954	53.06122		
1.706	94.88726						

Figure 6 D

HA beads pulldown and
Anti-FLAG antibody detection

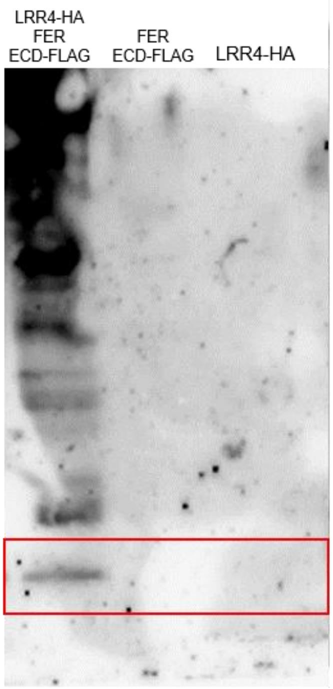

Anti-HA total protein extract
detection

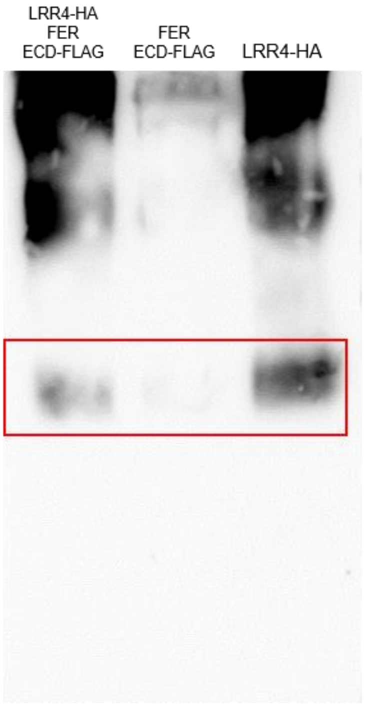

Anti-FLAG total protein extract
Detection (12% gel)

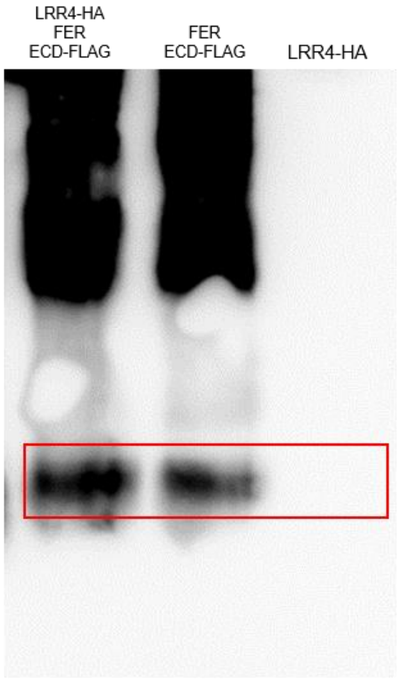

Supplement: Supplementary file 13 — Source Data for Figure 6 [file EMBJ-38-e100353-s011.pdf]
