# Supplementary material for: Extracellular matrix sensing by FERONIA and Leucine‐Rich Repeat Extensins controls vacuolar expansion during cellular elongation in Arabidopsis thaliana
Source: EMBO J. 2019 Mar 8;38(7):e100353. doi: 10.15252/embj.2018100353 (PMC6443208; doi:10.15252/embj.2018100353)
Supplement: Supplementary file 14 — Source Data for Figure 7 [file EMBJ-38-e100353-s012.pdf]

Figure 7 B

Col-0						35S::LRR4-cit					
DMSO			EGCG			DMSO			EGCG		
		vac. morph. index			vac. morph. index			vac. morph. index			vac. morph. index
length	15.506		length	5.609		length	13.03		length	14.954	
width	8.34	129.32	width	5.142	28.84148	width	7.452	97.09956	width	7.005	104.7528
	11.929			5.547			10.979			17.794	
	8.603	102.6252		4.258	23.61913		8.211	90.14857		10.062	179.0432
	6.702			6.558			10.981			13.813	
	4.196	28.12159		4.947	32.44243		9.095	99.8722		10.607	146.5145
	6.628			4.251			8.105			11.595	
	5.142	34.08118		3.874	16.46837		7.703	62.43282		7.335	85.04933
	10.014			9.067			16.361			9.886	
	7.692	77.02769		4.739	42.96851		10.264	167.9293		7.005	69.25143
	11.076			8.942			15.424			9.863	
	6.05	67.0098		3.123	27.92587		11.076	170.8362		7.932	78.23332
	7.296			7.208			14.263			10.894	
	4.258	31.06637		4.59	33.08472		12.159	173.4238		8.603	93.72108
	5.63			6.732			16.793			14.184	
	3.753	21.12939		3.604	24.26213		11.744	197.217		5.479	77.71414
	11.775			11.834			10.715			10.64	
	5.811	68.42453		12.531	148.2919		9.246	99.07089		7.87	83.7368
	7.144			7.636			13.116			10.32	
	6.026	43.04974		6.126	46.77814		8.464	111.0138		6.796	70.13472
	3.911			6.021			17.018			15.394	
	2.643	10.33677		5.335	32.12204		11.19	190.4314		6.026	92.76424
	5.711			4.148			15.58			11.795	
	3.44	19.64584		3.612	14.98258		11.422	177.9548		8.173	96.40054
	12.016			6.611			15.137			19.855	
	7.748	93.09997		3.268	21.60475		12.977	196.4328		10.343	205.3603
	11.573			5.484			18.427			13.291	
	10.815	125.162		2.802	15.36617		9.274	170.892		7.506	99.76225
	8.44			3.911			13.217			12.916	
	6.487	54.75028		3.338	13.05492		12.263	162.0801		7.598	98.13577
	11.598			8.406			15.139			13.302	
	10.094	117.0702		7.541	63.38965		12.494	189.1467		7.692	102.319
	5.949			5.017			14.224			19.848	
	4.278	25.44982		3.372	16.91732		11.13	158.3131		12.878	255.6025
	5.372			8.532			10.615			14.434	
	3.011	16.17509		5.421	46.25197		11.812	125.3844		12.551	181.1611
	4.331			6.469			10.572			10.037	
	5.051	21.87588		4.092	26.47115		8.903	94.12252		5.63	56.50831
	8.059			8.183			17.396			9.044	
	3.415	27.52149		5.051	41.33233		11.595	201.7066		6.126	55.40354
	8.44			7.826			13.935			15.663	
	5.291	44.65604		5.846	45.7508		10.343	144.1297		11.861	185.7788
	15.708			3.911			12.974			13.954	
	5.291	83.11103		3.667	14.34164		10.815	140.3138		10.572	147.5217
	6.252			6.265			10.793			12.59	
	3.844	24.03269		3.398	21.28847		7.677	82.85786		9.383	118.132
	4.298			4.811			14.514			20.663	
	3.011	12.94128		3.675	17.68043		9.673	140.3939		13.697	283.0211
	11.414			6.469			13.386			14.752	
	8.34	95.19276		2.474	16.00431		9.423	126.1363		12.496	184.341
	11.832			3.058			13.354			18.914	
	12.443	147.2256		3.077	9.409466		11.315	151.1005		11.023	208.489
	11.545			3.372			11.543			15.444	
	4.9	56.5705		2.972	10.02158		9.638	111.2514		6.492	100.2624
	8.812			6.126			9.529			9.886	
	4.278	37.69774		3.761	23.03989		4.805	45.78685		12.403	122.6161
	12.121			7.826			11.2			12.77	
	9.468	114.7616		4.028	31.52313		5.051	56.5712		10.117	129.1941
	10.334			11.498			17.341			7.703	
	7.307	75.51054		4.622	53.14376		12.275	212.8608		5.372	41.38052
	7.452			5.399			15.766			12.645	
	3.364	25.06853		3.011	16.25639		10.432	164.4709		11.625	146.9981
	7.521			3.465			15.089			8.13	
	3.338	25.1051		3.347	11.59736		11.753	177.341		5.484	44.58492
	13.979			4.733			17.142				
	12.073	168.7685		2.686	12.71284		13.729	235.3425			
	16.928			2.215			14.68				
	8.059	136.4228		2.267	5.021405		8.299	121.8293			
	9.568			5.091			10.094				
	7.666	73.34829		2.508	12.76823		9.665	97.55851			
	9.143						9.441				
	5.017	45.87043					8.709	82.22167			

Figure 7 C

Col-0

control		0.5 µM		0.75 µM		1 µM	
absolute	relative	absolute	relative	absolute	relative	absolute	relative
1.983	91.85195	2.021	93.61209	1.93	89.397	1.063	49.23783
2.251	104.2656	1.928	89.30436	1.56	72.25872	1.211	56.09314
2.171	100.56	1.918	88.84117	1.355	62.76318	1.24	57.43642
2.415	111.8621	1.955	90.55499	1.24	57.43642	1.16	53.73084
2.1	97.27135	1.62	75.0379	1.487	68.87738	1.074	49.74735
1.992	92.26882	1.766	81.80057	1.616	74.85262	1.073	49.70103
2.201	101.9496	1.747	80.9205	1.603	74.25046	1.274	59.01129
2.22	102.8297	1.815	84.07024	1.497	69.34058	1.217	56.37106
2.292	106.1647	1.842	85.32087	1.467	67.95099	1.317	61.00303
2.163	100.1895	1.68	77.81708	1.459	67.58043	1.074	49.74735
1.96	90.78659	2.096	97.08607	1.802	83.46808	1.354	62.71686
		1.875	86.84942	1.512	70.03537	1.247	57.76065
				1.659	76.84437		

35S::LRR4-cit #2

control		0.5 µM		0.75 µM		1 µM	
absolute	relative	absolute	relative	absolute	relative	absolute	relative
2.167	112.391	1.488	77.17478	0.95	49.27154	0.693	35.94229
2.173	112.7022	1.207	62.60078	1.027	53.26512	0.67	34.7494
2.123	110.1089	1.182	61.30416	0.905	46.93762	0.638	33.08973
1.975	102.4329	1.245	64.57164	0.979	50.77561	0.643	33.34905
2.015	104.5075	1.225	63.53435	1.066	55.28785	0.67	34.7494
1.884	97.71323	1.197	62.08213	1.05	54.45801	0.734	38.06874
1.782	92.42303	1.168	60.57806	1.015	52.64275	0.573	29.71852
2.11	109.4347	1.07	55.49531	0.986	51.13867	0.616	31.9487
1.467	76.08562	1.268	65.76453	1.064	55.18412	0.749	38.84672
1.973	102.3292	1.138	59.02211	1.024	53.10953	0.7	36.30534
1.54	79.87175	1.255	65.09029	1.05	54.45801	0.678	35.16432
		1.309	67.89099				
		1.23	63.79367				
		1.203	62.39332				
